# Feasibility and coexistence of large ecological communities

**DOI:** 10.1038/ncomms14389

**Published:** 2017-02-24

**Authors:** Jacopo Grilli, Matteo Adorisio, Samir Suweis, György Barabás, Jayanth R. Banavar, Stefano Allesina, Amos Maritan

**Affiliations:** 1 Department of Ecology and Evolution, University of Chicago, Chicago, Illinois 60637, USA; 2 International School for Advanced Studies (SISSA), via Bonomea 265, I-34136 Trieste, Italy; 3 Department of Physics and Astronomy ‘Galileo Galilei’, Università degli Studi di Padova, INFN and CNISM, Padova 35131, Italy; 4 Department of Physics, University of Maryland, College Park, Maryland 20742, USA; 5 Computation Institute, University of Chicago, Chicago, Illinois 60637, USA; 6 Northwestern Institute on Complex Systems, Northwestern University, Evanston, Illinois 60208, USA

## Abstract

The role of species interactions in controlling the interplay between the stability of ecosystems and their biodiversity is still not well understood. The ability of ecological communities to recover after small perturbations of the species abundances (local asymptotic stability) has been well studied, whereas the likelihood of a community to persist when the conditions change (structural stability) has received much less attention. Our goal is to understand the effects of diversity, interaction strengths and ecological network structure on the volume of parameter space leading to feasible equilibria. We develop a geometrical framework to study the range of conditions necessary for feasible coexistence. We show that feasibility is determined by few quantities describing the interactions, yielding a nontrivial complexity–feasibility relationship. Analysing more than 100 empirical networks, we show that the range of coexistence conditions in mutualistic systems can be analytically predicted. Finally, we characterize the geometric shape of the feasibility domain, thereby identifying the direction of perturbations that are more likely to cause extinctions.

## Abstract (web summary)

A central question in theoretical ecology is how diverse species can coexist in communities, and how that coexistence depends on network properties. Here, Grilli *et al*. quantify the extent of feasible coexistence of empirical networks, showing that it is smaller for trophic than mutualism networks.

Natural populations are faced with constantly varying environmental conditions. Environmental conditions affect physiological parameters (for example, metabolic rates^1^) as well as ecological ones (for example, the presence and strength of interactions between populations^2^,^3^,^4^,^5^). Therefore, in order to persist, ecological communities necessarily need, at the very least, to be able to cope with small environmental changes. Mathematically, this translates into an argument on the robustness of the qualitative behaviour of an ecological dynamical system: to guarantee robust coexistence, a model describing an ecological community needs at least to be (qualitatively) insensitive to small perturbations of the parameters^6^,^7^. This notion has been formalized in the measure of robustness^8^ or structural stability^9^, expressed as the volume of the parameter space resulting in the coexistence of all populations in a community.

While the local asymptotic stability (the ability to recover after a small change in the population abundances) of ecological communities has been studied in small^10^ and large^11^,^12^,^13^,^14^ systems, the study of structural stability (that is, the ability of a community to retain the same dynamical behaviour if conditions are slightly altered)—despite being proposed early on as a key feature in the context of the diversity–stability debate^15^,^16^,^17^,^18^—has historically been restricted to the case of small communities, with the first studies of larger communities appearing only recently^9^,^19^, and—because of mathematical limitations—dealing exclusively with the case of large mutualistic communities. Studies of structural stability have so far focused on the effect of ecological network structure (who interacts with whom) on the volume of parameter space leading to feasible equilibria, in which all populations have positive abundances.

Here we develop a geometrical framework for studying the feasibility of large ecological communities. We overcome the limitations that have hitherto prevented the study of consumer–resource networks, thereby providing a unified view of feasibility in ecological systems. Using a random matrix approach (which helped identify main drivers of local asymptotic stability), we pinpoint the key quantities controlling the volume of parameter space leading to feasible communities, as well as its sensitivity to changes in these parameters. We then contrast these expectations for randomly connected systems with simulations on structured empirical networks, quantifying the effects of network structure on feasibility.

## Results

### Theoretical framework

For simplicity, we consider a community composed of *S* species whose dynamics is determined by a system of autonomous ordinary differential equations:


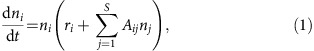


where *n*_*i*_ is the density of population *i*, *r*_*i*_ is its intrinsic growth rate and *A*_*ij*_ measures the interaction strength between population *i* and *j*. In this paper we consider only the linear functional response (that is, ***A*** does not depend on ***n***). In Supplementary Note 13 we discuss how and under which condition one could generalize our results to nonlinear functional responses. A fixed point n* (that is, a vector of densities making the right side of each equation zero) is feasible if 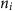
*>0 for every population. A fixed point is locally asymptotically stable if, following any sufficiently small perturbation of the densities, the system returns to a small vicinity of the fixed point. The fixed point is globally asymptotically stable if the system eventually return to it, starting from any positive initial condition within a finite domain. A system with a fixed point is structurally stable if, following a sufficiently small change in the growth rates *r*_*i*_, the new fixed point is still feasible and stable.

To study the range of conditions leading to stable coexistence, we need to disentangle feasibility and local stability. This problem is well discussed in ref. 9, where it was solved for the case of one possible parameterization of mutualistic interactions. If ***A*** is diagonally stable or Volterra-dissipative (that is, there exists a positive diagonal matrix ***D*** such that ***DA***+***A***^*T*^***D*** is stable), then any feasible fixed point is globally stable^20^,^21^. Unfortunately, a general characterization of this class of matrices is unknown^22^. We proceeded therefore by considering only the matrices such that all the eigenvalues of ***A***+***A***^*T*^ are negative (that is, the matrix ***A*** is negative definite in a generalized sense^23^, corresponding to ***D*** being equal to the identity matrix; see Methods and Supplementary Notes 1 and 2). This choice reduces the number of parameterizations one can analyse, as not all the diagonally stable matrices are negative definite. However, as shown in Supplementary Fig. 1, only very few parameter combinations are excluded from this set. Moreover, the effects of negative definitness are well studied for random matrices^24^, and by using it we can extend the study of feasibility to any ecological network, including food webs.

**Figure 1:**
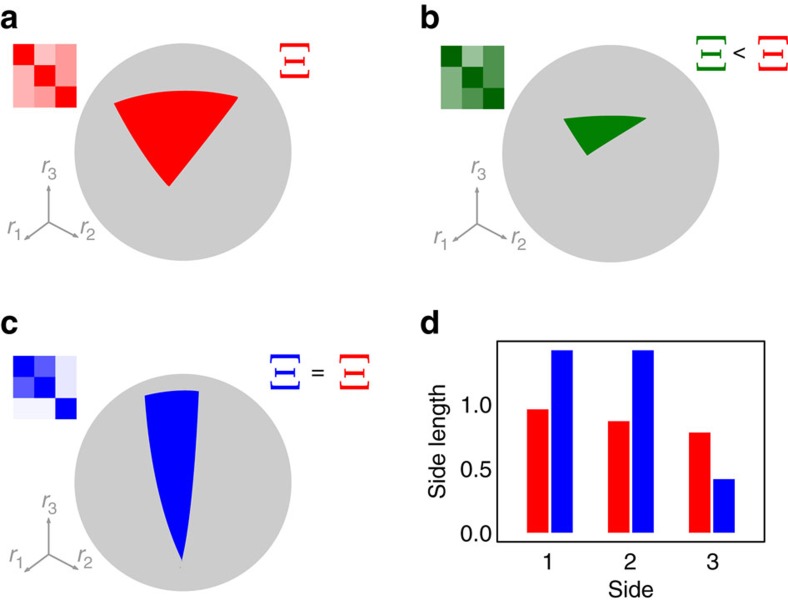
Geometrical properties of feasibility. The panels show the size and shape of the feasibility domain for three interaction matrices, each defining the interactions between three populations. If ***r*** corresponds to a feasible equilibrium, so does *c**r*** for any positive *c*; one can therefore study the feasibility domain on the surface of a sphere^25^ (Supplementary Note 3). The grey sphere represents the *S*=three-dimensional space of growth rates, while the coloured part corresponds to the combination of growth rates leading to stable coexistence. The area (or volume for higher-dimensional systems) of the coloured part is measured by Ξ. Larger values of Ξ correspond to a higher fraction of growth rate combinations leading to coexistence: the red interaction matrix (panel **a**) is therefore more robust against perturbations of ***r*** than the green one (**b**). The size of this region (that is, the value of Ξ) does not capture all the properties relevant for coexistence. The red (**a**) and blue (**c**) systems have the same Ξ, but the two regions—despite having the same area—have very different shapes, summarized in **d**, where we show the length of each side for the red and blue systems. In the red system (**a**), the three sides have about the same length, and thus moving from the centre in any direction will have about the same effect. In the blue system (**c**), however, one side is much shorter than the other two, implying that even small perturbations falling along this direction may drive the system outside the feasibility domain. One of our main results is that, roughly speaking, if the red system corresponds to the random case, then the green one to food webs (having the same heterogeneity in side lengths as the random case but with a smaller Ξ overall), and the blue one to empirical mutualistic networks (Ξ rougly the same as in the random case but with the heterogeneity in side lengths much greater).

Our goal is to measure the fraction of growth rate combinations, out of all possible ones, that lead to the coexistence of all *S* populations. Since we can separate stability and feasibility, we only need to find those *r*_*i*_ leading to feasible fixed points, and the condition above ensures that these will be globally stable. As pointed out before^9^, the problem is not to find a particular set of *r*_*i*_ leading to coexistence, but rather to measure how flexibly one may choose these rates. As shown in Fig. 1, this quantity—indicated by Ξ henceforth—can be thought of as a volume, or more precisely a solid angle, in the space of growth rates^25^ (see and Supplementary Note 3).

To calculate Ξ, one might naively wish to perform direct numerical computation of the fraction of growth rates, leading to a feasible equilibrium. While a direct calculation is viable when *S* is sufficiently small, this procedure becomes extremely inefficient for large *S* (ref. 9). We introduce a method that can be used to efficiently calculate Ξ with arbitrary precision, even for large *S* (see Supplementary Note 4). Using this method, we can accurately measure the size of the feasibility domain, with larger values of Ξ corresponding to larger proportions of conditions (intrinsic growth rates) compatible with stable coexistence. For reference, we normalize Ξ so that Ξ=1 when populations are self-regulated and not interacting (Methods), that is, when the interaction matrix ***A*** is a negative diagonal matrix, and thus equation (1) simplifies to *S* independent logistic equations.

### Feasibility is universal for large random matrices

May’s seminal work^11^ pioneered the use of random matrices as a reference, or null model, of ecological interactions. A particularly interesting feature of random matrices is that the distribution of their eigenvalues (determining local stability) is universal^26^. This means that local stability depends on just a few, coarse-grained properties of the matrix (that is, the number of species and the first few moments of the distribution of interaction strengths) and not on the finer details (for example, the particular distribution of interaction strengths; see Supplementary Note 5). In fact, these moments can be combined into just three parameters: *E*_1_, *E*_2_ and *E*_*c*_ (Methods). Together with *S*, they completely determine local asymptotic stability.

We tested whether universality also applies to feasibility. We considered different random matrix ensembles obtained for different connectance values and distributions from which the matrix entries were drawn, but with constant values of *S* and of *E*_1_, *E*_2_ and *E*_*c*_. We then checked whether the size Ξ of the feasibility domain depended only on these four quantities or also on finer details. Surprisingly, we found that the feasibility of random matrices is also universal (Methods Supplementary Note 5 and Supplementary Fig. 2). Two very different (random) ecosystems, with completely different interaction types and distributions of interaction strengths, but having the same number of species *S* and the same *E*_1_, *E*_2_ and *E*_*c*_, have the same Ξ in the large *S* limit. This result has important theoretical implications, as it indicates those moments as the drivers of feasibility, but also very practical consequences, namely that the parameter space one needs to explore is dramatically reduced.

### An analytical complexity–feasibility relationship

**Figure 2:**
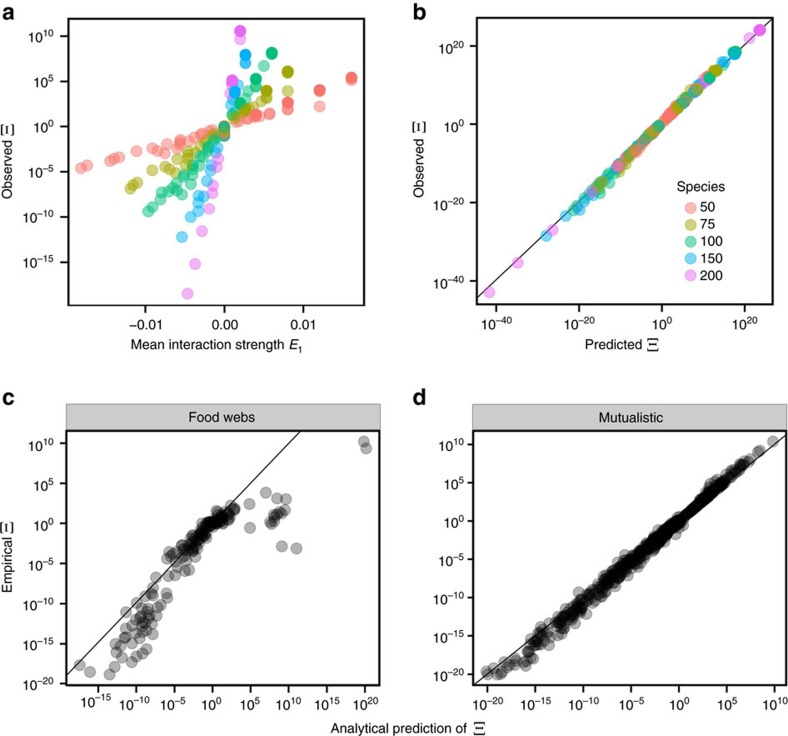
Feasibility domain in random and empirical webs. The top two panels show Ξ, the size of the domain of growth rates leading to coexistence, in the case of random networks. (**a**) The dependence of Ξ on *E*_1_=*Cμ* (where *C* is the connectance and *μ* is the mean interaction strength), and the number of species *S*. (**b**) The match between our analytical prediction (equation (2) and Supplementary Note 6) and the numerical value of Ξ. The bottom panels show a comparison between Ξ computed for empirical webs (89 mutualistic networks in **d**, and 15 food webs in **c**, parameterized with different distributions of interaction strengths) and our analytical approximation. Mutualistic networks have values of Ξ comparable to random networks with similar interactions (*R*^2^=0.98), indicating that their structure has little effect on the size of the feasibility domain. Food webs have lower values of Ξ than their random counterparts (*R*^2^=0.80). Empirical networks were parameterized extracting interaction strengths from a bivariate normal distribution with different means, variances and correlations (see and Supplementary Note 8).

The universality of Ξ suggests that it is amenable to analytical treatment. As explained in Supplementary Note 6 and shown in Fig. 2, when the mean and variance of interaction strengths are not too large and in the limit of large number of species, we are able to derive the following approximation for Ξ for large random interaction matrices ***A***:


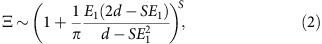


where *S* is is the number of species, *d* is the mean of ***A***’s diagonal entries and *E*_1_=*Cμ*, the product of the connectance *C* and the average interaction strength *μ* (see Methods). A more accurate formula is presented in Supplementary Note 6.

In analogy with the celebrated result of May^11^ connecting stability and complexity, equation (2) can be considered as a complexity–feasibility relationship. While in May’s scenario and in its generalizations^12^ the effect of complexity and diversity on stability is always detrimental, it does depend on the interaction type in the case of feasibility. Given that *d* is negative by construction, having more species or connections can either increase (*E*_1_>0) or shrink (*E*_1_<0) the size of the feasibility domain, as a function of the sign of interaction strenghts (see Fig. 2). It is important to stress that we computed Ξ under the assumption of ***A*** being negative definite. When we consider how Ξ depends on *S* and other parameters, we need to take into account the conditions making the matrix negative definite (see Methods and Supplementary Notes 2 and 5). In the case of positive interaction strengths, this condition is *d*+*SCμ*<0, implying an upper bound for *μ* that depends on *S*.

### Analytical prediction of the feasibility of empirical networks

Having explored the feasibility of random networks, we proceed to investigate the effects of incorporating empirical network structure. Ecological networks are in fact non-random^27^,^28^,^29^, and many studies have hypothesized that the structure of interactions could increase the likelihood of coexistence^30^,^31^,^32^. Having an analytical prediction for random matrices, we can study whether it predicts the size of the feasibility domain for empirical networks as well. Figure 2 shows the simulated values of Ξ for 89 mutualistic networks and 15 food webs (Supplementary Note 8 and Supplementary Table 1), parameterized multiple times and compared with our analytical approximation (see Methods). We find that Ξ of empirical mutualistic networks is well predicted by our formula, while it overestimates the feasibility domain of food webs, indicating that their non-random structure has a strong negative effect on feasibility.

We compared the effect of the empirical structure of mutualistic networks with randomizations, by controlling for the interaction strengths (see Supplementary Note 9 and Supplementary Figs 5–9). We show that, in the absence of variability in interaction strengths, the structure of empirical mutualistic networks has a positive effect on feasibility, which is strongly reduced when interaction strengths are allowed to vary. While this effect of empirical mutualistic networks is statistically significant, its effect on Ξ is negligible compared with the effect of the mean interaction strengths, and can only be detected by controlling very precisely for interaction strengths (see Supplementary Note 9). On a broader scale, as shown in Fig. 2, the size of the feasibility domain of empirical networks is well predicted by our analytical formula.

On the other hand, the negative effect of food web structure on Ξ is substantial. We compare each network with randomizations and also with predictions of the cascade model^27^, which has recently been shown to predict well the stability of empirical food webs^14^ (see Supplementary Note 9 and Supplementary Figs 10–12). By analysing different parameterizations we found that the feasibility domain of empirical structures is consistently and significantly smaller than that of both the randomizations and the cascade model. For most of the webs, the prediction obtained from the cascade model is better than that of randomizations, suggesting that the directionality of empirical webs plays a role in reducing feasibility, with other properties of the structure of empirical networks also contributing significantly to feasibility.

### Shape of the feasibility domain

So far, we have focused on the volume of the parameter space resulting in feasiblity. However, two systems having the same Ξ can still have very different responses to parameter perturbations, just as two triangles having the same area need not to have sides of the same length (Fig. 1). The two extreme cases correspond to (a) an isotropic system in which if we start at the barycentre of the feasibility domain, moving in any direction yields roughly the same effect (equivalent to an equilateral triangle); (b) anisotropic systems, in which the feasibility domain is much narrower in certain directions than in others (as in a scalene triangle). For our problem, the domain of growth rates leading to coexistence is—once the growth rates are normalized—the (*S*−1)-dimensional generalization of a triangle on a hypersphere. For *S*=3, this domain is indeed a triangle lying on a sphere as shown in Fig. 1. If all the *S*(*S*−1)/2 sides of this (hyper-)triangle are about the same length, then different perturbations will have similar effects on the system. On the other hand, if some sides are much shorter than others, then there will be changes of conditions which will more likely have an impact on coexistence than others. We therefore consider a measure of the heterogeneity in the distribution of the side lengths (Fig. 1 and Supplementary Note 10). The larger the variance of this distribution, the more likely it is that certain perturbations can destroy coexistence, even when Ξ is large and the perturbation small. This way of measuring heterogeneity is particularly convenient because it is independent of the initial conditions. Moreover, the length of each side can be directly related to the similarity between the corresponding pair of species (Supplementary Note 10), drawing a strong connection between the parameter space allowing for coexistence and the phenotypic space. As in the case of Ξ, this measure is a function of the interaction matrix and corresponds to a geometrical property of the coexistence domain.

**Figure 3:**
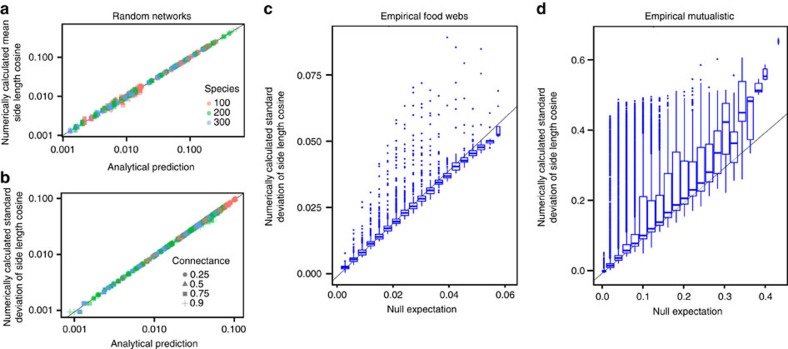
Distribution of side lengths in random and empirical networks. Left panels show the mean (**a**) and the s.d. (**b**) of cos(*η*), where *η* is the side length. Analytical predictions for the first two moments of cos(*η*) (Supplementary Note 10) perfectly match the numerical simulations. The two panels on the right show the s.d. of cos(*η*) for food webs (**c**) and mutualistic networks (**d**) compared with the expectations for the randomized cases. Both trophic and mutualistic interactions show larger fluctuations of side lengths, suggesting the existence of perturbation directions to which the system is more sensitive than to others. This effect is particularly pronounced and relevant for mutualistic networks. While mutualistic and random networks have a similar feasibility domain size Ξ, this result implies that the response of mutualistic networks to perturbations is in fact more heterogeneous than those of their random counterparts.

While Ξ is a universal quantity for random networks, the distribution of side lengths is not: it depends on the full distribution of interaction strengths (Supplementary Note 10). On the other hand, it is possible to compute it analytically in full generality, that is, for any distribution of interaction strengths and any interaction types. In particular, we are able to obtain an expression for its mean and variance, which depend only on *S*, *E*_1_, *E*_2_ and *E*_*c*_ (Supplementary Note 10). Figure 3 shows that the analytical formula, in the case of random ***A***, matches the observed mean and variance of side lengths of random networks perfectly.

### Empirical feasibility domains have more heterogeneous shapes

As we have done for Ξ, we can now test how non-random empirical network topologies influence the distribution of side lengths. Figure 3 shows that empirical food webs and, in particular, empirical mutualistic networks display a much larger variation in side lengths than expected by chance. This result is particularly relevant, indicating that even if the feasibility domains of empirical mutualistic networks are equal or larger than those of random networks, their shapes are less regular than expected by chance, and thus we expect perturbations in certain directions to quickly lead out of the feasible domain of growth rates.

## Discussion

A classic problem in mathematical ecology is determining the response of systems to perturbations of model parameters. In the community context, one important application is getting at the range of parameters allowing for species coexistence^33^,^34^,^35^. Several methods exist for evaluating this range^7^,^36^,^37^,^38^, but they either rely on raw numerical techniques or else can only evaluate system response to small parameter perturbations. Here in the context of the general Lotka–Volterra model, we have given a method for the global assessment of all combinations of species’ intrinsic growth rates compatible with coexistence—what we have called the domain of feasibility. Our geometrical approach can determine not only the total size of the feasibility domain, but also its shape: it is always a simply connected domain forming a convex polyhedral cone whose side lengths can be evaluated from the interaction matrix. Applying our method to empirical interaction networks, we were able to characterize the region of parameter space compatible with coexistence; the importance of this kind of information is underlined by a rapidly changing environment that is expected to cause substantial shifts in the parameters influencing these systems.

The geometrical framework we employed, pioneered by Svirezhev and Logofet^25^, allows for the formulation of a complexity–feasibility relationship. In analogy with the celebrated complexity–stability relationship, it relates the size of the feasibility domain with diversity, connectance and interaction strengths of a random interacting community. While communities are not random, this relationship sets a null expectation for the scaling of the proportion of feasible conditions. We obtain that the mean of interaction strengths sets the behaviour of feasibility with the number of species. If the mean is negative (for example, in case of competition or predation with limited efficiency), the larger the system is, the smaller is the set of conditions leading to coexistence, while for positive mean (for example, in the case of mutualism) the converse is true.

Here we have shown that the fraction of conditions compatible with coexistence is mainly determined by the number and the mean strength of interactions. In terms of network properties, the relevant quantity is the connectance, with other properties (for example, nestedness or degree distribution) having minimal effects. In particular, once the connectance and mean interaction strength are fixed, the matrices built using empirical mutualistic networks have feasibility domains very similar to that expected for the random case, as was also observed previously in a similar context^39^.

The empirical network structure of mutualistic networks has a statistically significant effect on the size of the feasibility domain. Whether this effect is ecologically relevant depends on the specific application at hand. For instance, the effect of structure could be neglected to quantify how the feasibility domain would change if a fraction of pollinators went extinct, and it could be evaluated using our analytical result. In contexts where the interaction strengths are strongly constrained, structure would play an important role. Our method provides, in this respect, a direct way of quantifying the importance of different factors, disentangling the way different interaction properties affect feasibility.

For mutualistic interaction networks, our results clearly show which properties determine the global health of the community, and therefore indicate which properties should be measured in the field. While not observing a link or measuring a wrong interaction coefficient could have strong effects on ecosystem dynamics, they have very little effect on the size of the feasibility domain and how the community copes with environmental perturbations and how likely extinctions are^40^. The major role is played by corse-grained statistical properties of the interactions, such as connectance or the mean and variance of the interaction strengths.

For food webs, on the other hand, empirical systems tend to have feasibility domains smaller than either their random counterparts or models conserving the directionality of interactions (cascade model). It is an open question which properties of real food webs are responsible for restricting the feasibility domain in this way. A possible candidate is the group structure observed in food webs^41^, corresponding to larger similarity of how certain species interact with the rest of the system than expected by chance, which in turn reduces the size of the feasibility domain.

These results parallel those for the distribution of the side lengths of the convex polyhedral cone delimiting the feasibility domain. The variance of side lengths for empirical structures is much higher than that in random networks. This implies that even if the total size of the feasibility domain is large, it will have a distorted shape that is very stretched along some directions and shortened along others (Fig. 1). Consequently, it will be possible to find growth rate perturbations of small magnitude that will drive the system outside its feasibility domain^42^.

We have shown that each side of the feasibility domain corresponds to a pair of species, with the length determined by how similarly the two species interact with the rest of the system. As two species interact more and more similarly (that is, have a larger niche overlap), the corresponding side becomes shorter and shorter, which in turns means greater sensitivity to parameter perturbations. Consistently with earlier results^7^,^8^, this fact establishes a relationship between niche overlap and the range of conditions that lead to coexistence: greater niche overlap means a more restricted parameter range allowing for coexistence, irrespective of the details of the interactions.

Several recent lines of work have studied the effect of network structure on coexistence in species-rich communities, with contrasting results^9^,^30^,^31^,^32^,^43^,^44^. For instance, on one hand nestedness was shown to increase biodiversity^31^, while, on the other hand, it is known to be associated with lower stability^43^,^45^. The differences between the size and shape of the feasibility domain shed light on these contrasting results. Most of these studies rely on numerical integration, and therefore strongly depend on initial conditions. Given the difference in the shape of the feasibility domains of random and empirical networks, different initial conditions and their perturbations could result in markedly different outcomes: the feasibility domain could appear to be large or small depending on the direction in which perturbations are made.

Our characterization of the geometrical properties of the feasibility domain contributes to the complete picture of the relation between feasibility and stability. It has been recently proposed that nestedness promotes larger feasibility domain sizes over stability^19^, suggesting the existence of a trade-off between feasibility and stability. As we showed, the (mild) increase of the feasibility domain size parallels with the increase of the variability of side lengths. The latter property is crucial to quantify the robustness to perturbations, and it might be interesting to explore more carefully the relation between stability and the shape of the feasibility domain.

Having established the general geometrical properties of the feasibility domain, we are in a much better position to critically evaluate the feasibility domains of real ecological communities. We consider this as a first step along the way of describing feasibility in more complex models and ecological scenarios.

## Methods

### Disentangling stability and feasibility

From equation (1), a feasible fixed point, if it exists, is given by the solution of


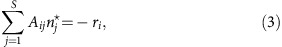


where the asterisk denotes equilibrium values. A fixed point is locally asymptotically stable if all eigenvalues of the community matrix


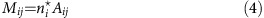


have negative real parts. As discussed in Supplementary Note 2, if ***A*** is diagonally stable or Volterra-dissipative (that is, there exists a positive diagonal matrix ***D*** such that ***DA***+***A***^*T*^***D*** is stable), then a feasible fixed point is globally stable in 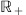.

A general characterization of diagonally stable matrices is unknown for more than three species^22^. There exist algorithms^46^ that reduce the problem of determining whether a *S* × *S* matrix is diagonally stable into two simultaneous problems of (*S*−1) × (*S*−1) matrices. While this method can be efficiently used to determine the diagonal stability of 4 × 4 matrices, it becomes computationally intractable for large *S*.

A matrix ***A*** is negative definite if


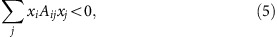


for any non-zero vector ***x***. A necessary and sufficient condition for a real matrix ***A*** to be negative definite is that all the eigenvalues of ***A***+***A***^*T*^ are negative^23^. A negative definite matrix is also diagonally stable, as the condition for diagonal stability holds with ***D*** being the identity matrix. Since it is extremely simple to verify this condition and it has been characterized for random matrices, we will study feasibility of negative definite matrices. In Supplementary Note 5 and Supplementary Fig. 2 we show that with this choice we are excluding only a small region of the parameter space.

### Size of the feasibility domain

The quantity Ξ is the proportion of intrinsic growth rates leading to feasible equilibria. While a more rigourous definition is presented in Supplementary Note 4, with a slight abuse of notation, Ξ can be thought of as


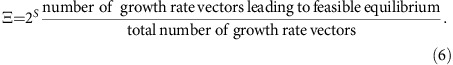


The factor 2^*S*^ is an arbitrary choice that does not affect the results. It has been introduced to have Ξ=1 in absence of interspecific interactions (*A*_*ij*_=0 if *i*≠*j* in equation (1)) and when all the species are self-regulated (*A*_*ii*_<0 if *i*≠*j* in equation (1)). Given the geometrical properties of the feasibility domain, the proportion of feasible growth rates can be calculated considering only growth rate vectors of length one (Fig. 1 and Supplementary Note 3), as this choice does not affect the value given by equation (6). In Supplementary Note 4 we provide an integral formula for Ξ (refs 47, 48), which makes both numerical and analytical calculations possible.

Our method is still valid if some of the species are not self-regulated (that is, *A*_*ii*_=0 for some *i*). In Supplementary Note 7 we explicitly discuss the properties of the feasibility domain of a community with consumer–resource interactions. In that case, Ξ=0 either when the diversity of consumers exceeds the diversity of resources or in the absence of interspecific interactions. Since consumers are regulated by their resources, they cannot survive in their absence and should therefore be characterized by negative intrinsic growth rates. We observe indeed that a necessary condition for an intrinsic growth rate vector to be contained in the feasibility domain is to have negative values for the components corresponding to consumers.

### Random matrices and moments

*E*_1_, *E*_2_ and *E*_*c*_ are moments of the random distribution for the off-diagonal elements of the interaction matrix, and are simply and directly related to the interaction strengths. They can be calculated as


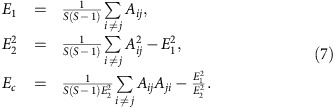


For random networks with connectance *C*, these expressions reduce to (ref. 26)


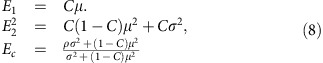


where *μ* is the mean of the interaction strengths, *σ* is their variance and *ρ* is the average pairwise correlation between the interaction coefficients of species pairs^26^.

### Universality of the size of the feasibility domain

The size of the feasibility domain should, at least in principle, depend on all the entries of the interaction matrix. When these elements are drawn from a distribution, the size Ξ of the feasibility domain is then expected to depend on all the moments of that distribution. As *S* increases, the dependence of Ξ on some of those moments and parameters might become less and less important. Ξ is universal if, in the limit of large *S*, it depends only on a few properties of the interaction matrix (that is, on just the first few moments of the distribution).

Specifically, for each unique pair of species (*i*, *j*), we set *A*_*ij*_=0 with probability 1−*C* and assign a random pair of interaction strengths (*M*_*ij*_, *M*_*ji*_)=(*x*, *y*) with probability *C*. The pair (*x*, *y*) is drawn from a bivariate distribution with given mean *μ*, variance *σ* and correlation *ρ* between *x* and *y* (ref. 26). By considering different bivariate distributions, we can analyse the effect of different sign patterns (for example, only (+, −) or (+, +) interactions) and different marginal distributions (for example, drawing elements from a uniform or a lognormal distribution).

Non-universality of Ξ would mean that it depends on all the fine details of the parameterization:


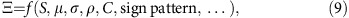


where *f*(·) is an arbitrary function. The dependence on *μ*, *σ* and *ρ* can, without loss of generality, be expressed in terms of *E*_1_, *E*_2_ and *E*_*c*_:


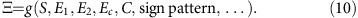


However, if Ξ is universal, then for large *S*, it is possible to express it as a function of *E*_1_, *E*_2_ and *E*_*c*_ only:


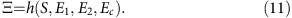


To verify this conjecture, we calculated Ξ for matrices with the same values of *E*_1_, *E*_2_ and *E*_*c*_ that differed for the values of the other parameters. As extensively shown in Supplementary Note 5, Ξ is uniquely determined by *S*, *E*_1_, *E*_2_ and *E*_*c*_ (equation (2)).

### Parameterization of mutualistic networks

The 89 mutualistic networks (59 pollination networks and 30 seed-dispersal networks) were obtained from the Web of Life data set (www.web-of-life.es), where references to the original works can be found. Empirical networks are encoded in terms of adjacency matrices ***L***: *L*_*ij*_=1 if species *j* interact with species *i* and 0 otherwise. When the original network was not fully connected, we considered the largest connected component.

In the case of mutualistic networks, the adjacency matrix ***L*** is bipartite, that is, it has the structure


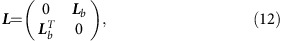


where ***L***_*b*_ is a *S*_*A*_ × *S*_*P*_ matrix (*S*_*A*_ and *S*_*P*_ being the number of animals and plants, respectively). The adjacency matrix contains information only about the interactions between animals and plants, but not about competition within plants or animals.

We parameterized the interaction matrix in the following way:


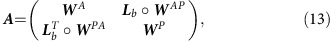


where the symbol o indicates the Hadamard or entrywise product (that is, (***A*** o ***B***)_*ij*_=*A*_*ij*_*B*_*ij*_), while ***W***^*A*^, ***W***^*AP*^, ***W***^*PA*^ and ***W***^*P*^ are all random matrices. ***W***^*A*^ and ***W***^*P*^ are square matrices of dimension *S*_*A*_ × *S*_*A*_ and *S*_*P*_ × *S*_*P*_, while ***W***^*AP*^ and ***W***^*PA*^ are rectangular matrices of size *S*_*A*_ × *S*_*P*_ and *S*_*P*_ × *S*_*A*_. The diagonal elements 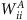 and 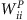 are set to −1, while the pairs (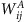, 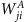) and (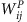, 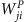) are drawn from a bivariate normal distribution with mean *μ*_−_, variance 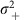
=*c*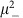 and correlation *ρ*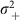. Since these two matrices represent competitive interactions, *μ*_−_<0. The pairs (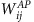, 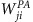) were extracted from a bivariate normal distribution with mean *μ*_+_, variance 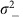
=*c*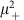, and correlation *ρ*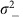, where *μ*_+_>0. For each network and parametrization we computed the size of the feasibility domain Ξ.

We considered different values of *μ*_−_, *μ*_+_, *c*, and *ρ*. Their values cannot be chosen arbitrarily, since ***A*** must be negative definite. For a choice of *c*, *ρ*, and a ratio *μ*_−_/*μ*_+_, the largest eigenvalue of (***A***+***A***^*T*^)/2 is linear in *μ*+(as an arbitrary *μ*_+_ can be obtained by multiplying *A* by *μ*_+_ and then shifting the diagonal). Given the values of *μ*_−_/*μ*_+_, *c* and *ρ*, one can therefore determine *μ*_max_, the maximum value of *μ*_+_ still leading to a negative definite ***A*** (that is, the value of *μ*_+_ such that the largest eigenvalue of (***A***+***A***^*T*^)/2 is equal to 0). Figure 2 was obtained by considering more than 1,000 parameterizations. Both the ratio *μ*_−_/*μ*_+_ and the coefficient of variation *c* could assume the values 0.5 or 2, while the correlation *ρ* assumed values from the set {−0.9, 0.5, 0, 0.5, 0.9}. The value of *μ*_+_ was set equal to 0.25*μ*_max_ and 0.75*μ*_max_. In addition, we considered the case *μ*_−_=0.

### Parameterization of food webs

In the case of food webs the adjacency matrix *L* is not symmetric, *L*_*ij*_=1 indicating that species *j* consumes species *i*. We removed all cannibalsistic loops. Since *L*_*ij*_ and *L*_*ji*_ are never simultaneously equal to one (there are no loops of length two), we parameterized the off-diagonal entries of ***A*** as


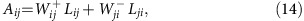


while the diagonal was fixed at −1. Both ***W***^+^ and ***W***^−^ are random matrices, where the pairs (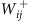, 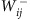) are drawn from a bivariate normal distribution with marginal means (*μ*_+_, *μ*_−_) and correlation matrix


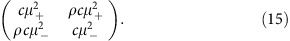


We considered considering different values of *μ*_−_, *μ*_+_, *c* and *ρ*. As explained above, given the values of *μ*_−_/*μ*_+_, *c* and *ρ*, one can determine *μ*_max_, the maximum value of *μ*_+_ still corresponding to a negative definite ***A***. Figure 2 was obtained by considering more that 350 parameterizations. Both the ratio *μ*_−_/*μ*_+_ and the coefficient of variation *c* could assume the values 0.5 or 2, while the correlation *ρ* assumed either the value −0.5 or 0.5. The value of *μ*_+_ was set either to 0.25*μ*_max_ or 0.75*μ*_max_.

### Data availability

The code needed to replicate the results presented here can be found at https://github.com/jacopogrilli/feasibility.

## Additional information

**How to cite this article:** Grilli, J. *et al*. Feasibility and coexistence of large ecological communities. *Nat. Commun.* **8,** 14389 doi: 10.1038/ncomms14389 (2017).

**Publisher’s note**: Springer Nature remains neutral with regard to jurisdictional claims in published maps and institutional affiliations.

## Supplementary Material

Supplementary Information Supplementary Figures, Supplementary Tables, Supplementary Notes and Supplementary References
