# Publisher Correction: Feasibility and coexistence of large ecological communities

**DOI:** 10.1038/ncomms16228

**Published:** 2018-07-13

**Authors:** Jacopo Grilli, Matteo Adorisio, Samir Suweis, György Barabás, Jayanth R. Banavar, Stefano Allesina, Amos Maritan

Nature Communications 8: Article number: 14389; DOI: 10.1038/ncomms14389; Published: 02 24 2017; Updated: 07 13 2018

The original HTML version of this Article had an incorrect article number of ‘0’; it should have been ‘14389’. This has now been corrected in the HTML version of the Article. The PDF version was correct from the time of publication.

