# Supplementary material for: Feasibility and coexistence of large ecological communities
Source: Nat Commun. 2017 Feb 24;8:14389. doi: 10.1038/ncomms14389 (PMC5333123; doi:10.1038/ncomms14389)
Supplement: Supplementary Information — Supplementary Figures, Supplementary Tables, Supplementary Notes and Supplementary References [file ncomms14389-s1.pdf]

Feasibility and coexistence of large ecological networks: a geometrical approach
Supplementary Notes

Supplementary Note 1 Community dynamics, feasibility, and stability

We consider an ecological community composed of S populations, whose dynamics is described by the following equations:

$$\frac{\mathrm{d}n_i}{\mathrm{d}t} = n_i \left(r_i + \sum_{j=1}^{S} A_{ij} n_j \right) , \tag{1}$$

where n_i is the population abundance of species i and r_i is its intrinsic growth rate, and A_{ij} is the effect of a unit change in species j's density on species i's per capita growth rate. For notational convenience, we collect the coefficients A_{ij} into the interaction matrix $\boldsymbol{A}$, and n_i and r_i into the vectors $\boldsymbol{n}$ and $\boldsymbol{r}$, respectively.

In principle, the interaction matrix $\boldsymbol{A}$ may depend on $\boldsymbol{n}$. We discuss this more general case in section Supplementary Note 13. In the following, we consider the simpler case of $\boldsymbol{A}$ being independent of $\boldsymbol{n}$; then, equation (1) is a general system of Lotka–Volterra population equations.

A vector $\boldsymbol{n}^*$ is a fixed point (equilibrium) if

$$0 = n_i^* \Big(r_i + \sum_{j=1}^{S} A_{ij} n_j^* \Big) \qquad (i = 1, 2, \ldots, S) . \tag{2}$$

A fixed point is feasible if $n_i^* > 0$ for all i. A feasible fixed point (if it exists) is then a solution to the equation

$$r_i = - \sum_{j=1}^{S} A_{ij} n_j^* , \tag{3}$$

and therefore, assuming $\boldsymbol{A}$ is invertible,

$$n_i^* = -\sum_{j=1}^{S} (A^{-1})_{ij} r_j \; . \tag{4}$$

A fixed point n_i^* is locally stable if the system returns to it following any sufficiently small perturbation of the population abundances. Introducing $n_i = n_i^* + \delta n_i$ in equation 1 and assuming that δn_i is small, we obtain, by expanding around $\delta n_i = 0$,

$$\frac{\mathrm{d}\delta n_i}{\mathrm{d}t} = \sum_{j=1}^{S} M_{ij} \delta n_j \; , \tag{5}$$

where M_{ij} is the (i, j)th entry of the Jacobian evaluated at the fixed point (also called the community matrix), which, in the case of equation 1, reduces to

$$M_{ij} = n_i^* A_{ij} = -\left(\sum_{k=1}^{S} (A^{-1})_{ik} r_k \right) A_{ij} \; . \tag{6}$$

Substituting into equation 5, we get

$$\frac{\mathrm{d}\delta n_i}{\mathrm{d}t} = -\sum_{j=1}^{S} \left(\sum_{k=1}^{S} (A^{-1})_{ik} r_k \right) A_{ij} \delta n_j \; . \tag{7}$$

There are two possible scenarios for the dynamics of equation 5. If all eigenvalues of $\boldsymbol{M}$ have negative real parts, then the perturbation $\delta \boldsymbol{n}$ decays exponentially to zero and n_i^* is locally stable. If at least one eigenvalue of $\boldsymbol{M}$ has a positive real part, then there exists an infinitesimal perturbation such that the system does not return to equilibrium. If we order the eigenvalues λ_i of $\boldsymbol{M}$ according to their real parts, i.e., $\Re(\lambda_1) > \Re(\lambda_2) > \cdots > \Re(\lambda_S)$, then stability depends exclusively on $\Re(\lambda_1)$: if it is negative, n_i^* is dynamically locally stable; otherwise, it is unstable [1].

A fixed point is globally stable if it is the final outcome of the dynamics from any initial condition involving strictly positive population abundances.

Supplementary Note 2 Disentangling stability and feasibility

As we can see from equations 4 and 7, both feasibility and stability depend on both $\boldsymbol{r}$ and $\boldsymbol{A}$ and, at least in principle, a fixed point can be stable or unstable, independently of the fact that it is feasible or not.

We want to study the proportion of conditions (i.e., the number of combinations of the growth rates $\boldsymbol{r}$ out of all possible combinations) leading to coexistence, i.e., leading to stable and feasible equilibria. Therefore in principle we should, for a fixed matrix $\boldsymbol{A}$, look for growth rates $\boldsymbol{r}$ that satisfy both stability and feasibility. In probabilistic terms, we want to measure the likelihood that a random combination of the intrinsic growth rates corresponds to a stable and feasible solution.

In the case of equation 1, it is possible to disentangle feasibility and stability by applying a mild condition on the interaction matrix $\boldsymbol{A}$. To this end, we introduce some terminology [2, section 2.1.2]:

- **Stability**. A real matrix $\boldsymbol{B}$ is stable if all its eigenvalues have negative real parts.
- ***D*-stability**. A real matrix $\boldsymbol{B}$ is D-stable if $\boldsymbol{D}\,\boldsymbol{B}$ is stable for any diagonal matrix $\boldsymbol{D}$ with strictly positive diagonal entries.
- **Diagonal stability**. A real matrix $\boldsymbol{B}$ is diagonally stable if there exists a positive diagonal matrix $\boldsymbol{D}$ such that $\boldsymbol{D}\,\boldsymbol{B} + \boldsymbol{B}^T\,\boldsymbol{D}$ is stable (where $\boldsymbol{B}^T$ is the transpose of $\boldsymbol{B}$).

We also consider

- **Negative definiteness** (in a generalized sense). A real matrix $\boldsymbol{B}$ is negative definite if

$\sum_{ij} x_i B_{ij} x_j < 0$ for any non-zero vector $\boldsymbol{x}$ [3].

These properties are closely related to each other [2, 4]:

$$\text{Negative definiteness} \implies \text{Diagonal stability} \implies D\text{-stability} \implies \text{Stability} \tag{8}$$

- **Negative definiteness $\implies$ Diagonal stability**. A matrix $\boldsymbol{B}$ is negative definite if and only if all the eigenvalues of $\boldsymbol{B} + \boldsymbol{B}^T$ are negative [3]. If this condition hold, then the positive diagonal matrix satisfying the definition of diagonal stability is simply the identity matrix.
- **Diagonal stability $\implies$ *D*-stability**. See the book by Kaszkurewicz & Bhaya for the proof [2, lemma 2.1.4].
- ***D*-stability $\implies$ Stability**. This follows from the definition of D-stability when $\boldsymbol{D}$ is the identity matrix.

In the case of equation 1, those conditions applied to the matrix $\boldsymbol{A}$ are related to the stability of the system. One can use the definition of the community matrix (equation 6) to show that ***D*-stability of $\boldsymbol{A}$ implies the local asymptotic stability of *any* feasible fixed point**. This is because the community matrix with entries $M_{ij} = n_i^* A_{ij}$ can be written as $\boldsymbol{N}\,\boldsymbol{A}$, where $\boldsymbol{N}$ is the diagonal matrix with $N_{ii} = n_i^*$. If the fixed point is feasible and $\boldsymbol{A}$ is D-stable, then local asymptotic stability is guaranteed. Moreover it is possible to show [5, 6] that **diagonal stability of $\boldsymbol{A}$ $\implies$ global stability**.

Thus, we have a condition on $\boldsymbol{A}$ that makes it possible to disentangle the problems of stability and feasibility: **$\boldsymbol{A}$ is negative definite $\implies$ global stability of the feasible fixed point** [7]. Therefore, if we assume $\boldsymbol{A}$ is negative definite, then feasibility of the equilibrium is sufficient

to guarantee its global stability as well, i.e., feasibility guarantees globally stable coexistence. Consistently with this, it is known that the largest eigenvalue of $(\boldsymbol{A} + \boldsymbol{A}^T)/2$ is always larger than or equal to the real part of $\boldsymbol{A}$'s leading eigenvalue [8], i.e. negative definiteness implies stability. While this was indeed observed before, it is important to underline that, in the case of ref. [8], this property was considered on the community matrix $\boldsymbol{M}$ (which also depends on the fixed point's position in phase space) and not on the interaction matrix $\boldsymbol{A}$.

Since we are interested in studying how interactions (i.e., the matrix $\boldsymbol{A}$) determine coexistence, and which properties of the former determine the latter, we will restrict our analysis to negative definite matrices $\boldsymbol{A}$ and focus only on the problem of feasibility. This condition has the advantage of being analytically computable for large random matrices (see section Supplementary Note 5.1).

Supplementary Note 3 Geometrical properties of the feasibility domain

In section Supplementary Note 2 we showed how to separate feasibility and stability, i.e., we have a sufficient condition on the interaction matrix that guarantees (global) stability of the feasible fixed point. The problem of determining the size of the coexistence domain is therefore reduced to that of determining the size of the feasibility domain. The ecological interpretation of this volume is the proportion of different conditions leading to feasible equilibria out of all possible conditions. The larger this volume is, the higher the probability that the system is able to sustain biodiversity. In terms of equation 1, we want to quantify the proportion of growth rate vectors $\boldsymbol{r}$ corresponding to a feasible fixed point.

This geometrical approach was pioneered in [1] where the space of feasible solution was

studied for dissipative systems, and the size of that domain was computed in the case $S = 3$ (see section Supplementary Note 12).

At this point, it is important to observe that if a vector $\boldsymbol{r}$ corresponds to a feasible solution, then $c\boldsymbol{r}$, c being an arbitrary positive constant, also corresponds to a feasible solution. This is because the equilibrium solution n_i^* is given by equation 4, which is linear in r_i. Therefore, the equilibrium corresponding to cr_i is simply cn_i^*, and since c is positive, cn_i^* is also feasible.

This fact implies that, given a large number of growth rate vectors $\boldsymbol{r}$, the expected proportion of vectors corresponding to a feasible fixed point is independent of $\boldsymbol{r}$'s norm. In other words, $\boldsymbol{r}$ is feasible if and only if $\boldsymbol{r}/\|\boldsymbol{r}\|$ is feasible, where $\|\boldsymbol{r}\| = \sqrt{\sum_i r_i^2}$ is the Euclidean norm of $\boldsymbol{r}$. The proportion of feasible growth rates among all possible ones is therefore equal to the proportion of feasible growth rates calculated using only growth rate vectors with $\|\boldsymbol{r}\| = 1$; i.e., those lying on the unit sphere.

Before proceeding with the mathematical definition of the size of the feasibility domain, we discuss the geometrical interpretation of equation 4. From this equation, the feasibility condition reads

$$\sum_{j=1}^{S} (A^{-1})_{ij} r_j < 0 \ . \tag{9}$$

This equation defines a convex polyhedral cone in the S-dimensional space of growth rates. A convex polyhedral cone [9] is a subset of $\mathbb{R}^S$ whose elements $\boldsymbol{x}$ can be written as positive linear combinations of N_G different S-dimensional vectors $\boldsymbol{g}^k$ called the generators of the cone:

$$\boldsymbol{x} = \sum_{k=1}^{N_G} \boldsymbol{g}^k \lambda_k \ , \tag{10}$$

where the λ_k are arbitrary positive constants. Due to this arbitrariness, if $\boldsymbol{g}^k$ is a generator of a given convex polyhedral cone, then also $c\boldsymbol{g}^k$ (where we rescale just the kth generator with the

positive constant c, leaving the others unchanged) will be a generator of the *same* cone [1]. In the case of equation 3, each and every growth rate vector belonging to the feasibility domain can be written as

$$r_i = -\sum_{k=1}^{S} A_{ik} n_k^* \ , \tag{11}$$

where, by definition, n_k^* is feasible and therefore a positive constant. One can easily see that this equation corresponds to equation 10 where the number of generators N_G is equal to S and the ith component of the vector $\boldsymbol{g}^k$ is proportional to $-A_{ik}$. As the lengths of the generators can be set to any positive value, we will normalize them to one, i.e.,

$$g_i^k(\boldsymbol{A}) = \frac{-A_{ik}}{\sqrt{\sum_{j=1}^{S}(A_{jk})^2}} \ . \tag{12}$$

The generators completely define the feasibility domain in the space of growth rates. A growth rate vector corresponds to a feasible equilibrium if and only if it can be written as a linear combination of the generators with positive coefficients. Biologically the generators correspond to the growth rate vectors that bound the coexistence domain. They correspond to nonfeasible equilibria with just one species with positive abundance (and all the others with zero abundance), such that there exist arbitrarily small perturbations of the growth rate vector that make the equilibrium feasible.

The set of all the growth rate vectors leading to a feasible equilibrium is therefore a convex polyhedral cone, defined by

$$K(\boldsymbol{A}) = \{\boldsymbol{r} \in \mathbb{R}^S | \sum_{j=1}^{S} (A^{-1})_{ij} r_j < 0\} \ . \tag{13}$$

Equivalently, it can be defined in terms of the generators:

$$K(\boldsymbol{A}) = \{\boldsymbol{r} \in \mathbb{R}^S | \exists \lambda_1, \lambda_2, \dots, \lambda_k > 0, \boldsymbol{r} = \sum_{k=1}^{S} \boldsymbol{g}^k(\boldsymbol{A}) \lambda_k\} \ , \tag{14}$$

where the generators $\boldsymbol{g}^k(\boldsymbol{A})$ are defined in equation 12. In section Supplementary Note 12 we show explicitly how these concepts pan out in the case of $S = 3$.

This geometrical definition and characterization of the feasibility domain allows us to identify classes of matrices having the exact same feasibility domain: they are simply matrices having the same set of generators. In particular, there are two basic transformations of the matrix $\boldsymbol{A}$ (and their combinations) that leave the set of generators unchanged: permutations and positive rescaling. A square matrix $\boldsymbol{P}$ is a permutation matrix if each row and column has one and only one nonzero entry and the value of that entry is equal to one. A positive rescaling is performed by a positive diagonal matrix $\boldsymbol{D}$. The set of generators of $\boldsymbol{A}$ is the same as those of $\boldsymbol{P}\,\boldsymbol{A}$ and $\boldsymbol{D}\,\boldsymbol{A}$. This can be seen by observing that a permutation of the rows just changes the order of the generators but not the generators themselves. In the same way, a generator with the same direction but different length generates the same cone, and so any positive constant that rescales a row of the matrix leaves the feasibility domain unchanged. It is important to note however that these two transformations do not leave the properties of the matrix $\boldsymbol{A}$ unchanged: both exchanging rows of a matrix and rescaling rows by different constants will in general change the structure of the matrix.

Using this geometrical framework, one can easily identify the center of the feasibility domain (also known as structural vector [6]). There are several possible ways to define the center of a hypervolume and, without additional assumptions, all the definitions are different. One natural choice is the barycenter ("center of mass") of the domain of feasible intrinsic growth rates. Any plane passing through the barycenter divides the volume into two subvolumes of equal size. The barycenter is equivalent to the center of mass of the volume (in the case of constant density). Then, the vector $\boldsymbol{x}^b$ pointing from the origin to the barycenter is given by

$$\boldsymbol{x}^b = \int_{K(A)\cap\mathbb{S}_S} \mathrm{d}^S\boldsymbol{y}\;\; \boldsymbol{y}\,, \tag{15}$$

where $\cap$ is the intersection of two sets, and $\mathbb{S}_S = \{\boldsymbol{r} \in \mathbb{R}^S | \|\boldsymbol{r}\| = 1\}$ represents the surface of the S-dimensional unit sphere. The variable $\boldsymbol{y}$ is therefore integrated over the feasibility do-

main restricted to the unit sphere's surface. All points in the feasibility domain are positive linear combinations of the generators, i.e.,

$$\boldsymbol{y} = \sum_k \lambda^k \boldsymbol{g}^k \ , \tag{16}$$

where the λ^k are positive constants. The fact that we consider only the points lying on the unit sphere, i.e., $\|\boldsymbol{y}\| = 1$, can be expressed as a constraint on $\boldsymbol{\lambda}$ (the vector of λs). Thus, we can write equation 15 as

$$\boldsymbol{x}^b = \int \mathrm{d}^S\boldsymbol{\lambda}\, q(\boldsymbol{\lambda}) \sum_k \lambda^k \boldsymbol{g}^k \ , \tag{17}$$

where q is an appropriate distribution, introduced to take into account three different constraints: all the components of $\boldsymbol{\lambda}$ must be positive; the vector $\sum_k \lambda^k \boldsymbol{g}^k$ must lie on the unit sphere; and those vectors must be sampled uniformly on the feasibility domain. One can show that the distribution $q(\boldsymbol{\lambda})$ has the following form

$$q(\boldsymbol{\lambda}) \propto \exp\left(-\sum_{i,j} \lambda^i (\boldsymbol{g}^i \cdot \boldsymbol{g}^j) \lambda^j\right) \prod_k \Theta(\lambda^k) \ , \tag{18}$$

where the proportionality constant is given by the normalization, the exponential term guarantees that the vectors $\sum_k \lambda^k \boldsymbol{g}^k$ are sampled uniformly on the sphere and the Heaviside function $\Theta(\lambda^k)$ constrains all the coefficients λ^k to be positive. Therefore, by defining,

$$\int \mathrm{d}^S\boldsymbol{\lambda}\, q(\boldsymbol{\lambda})\ \lambda^k =: \langle \lambda^k \rangle \ , \tag{19}$$

we obtain

$$\boldsymbol{x}^b = \sum_k \boldsymbol{g}^k \int \mathrm{d}^S\boldsymbol{\lambda}\, q(\boldsymbol{\lambda})\ \lambda^k = \sum_k \boldsymbol{g}^k \langle \lambda^k \rangle \ . \tag{20}$$

Supplementary Note 4 Definition and calculation of Ξ

As explained in section Supplementary Note 3, the proportion of feasible growth rates can be calculated considering only growth rate vectors of length one, i.e., $\|\boldsymbol{r}\| = 1$. This proportion

can be interpreted as the volume of the intersection of a convex cone and the surface of a sphere. Equivalently, it is the solid angle of the convex polyhedral cone [10, 11].

We define the quantity Ξ as

$$\Xi = 2^S \frac{\text{\# growth rate vectors corresponding to a feasible fixed point}}{\text{total \# growth rate vectors}} . \tag{21}$$

The factor 2^S that appears in this equation is an arbitrary choice, and it has been introduced to have $\Xi = 1$ when species are not interacting ($A_{ij} = 0$ if $i \neq j$). In this case equation 1 reduces to S independent logistic equations with equilibrium densities $n_i^* = -r_i/A_{ii}$. Taking each A_{ii} to be negative (otherwise each species would have an unstoppable positive feedback on itself), this equilibrium is feasible if and only if each r_i is positive. For a single species then, the probability of randomly drawing a feasible (i.e., positive) growth rate out of all possible growth rates is one half. For two species, both growth rates must have the correct sign to have the two species with positive abundance, and therefore the proportion of growth rate vectors satisfying this condition is $1/4$. For S species the combinations of the growth rates leading to a feasible fixed point is 2^{-S}. Ξ, defined as in equation 21, is therefore equal to one when species do not interact.

In terms of geometrical properties and the convex polyhedral cone, Ξ can be defined as

$$\Xi = 2^S \frac{\mathrm{vol}_{S-1}(K(A) \cap \mathbb{S}_S)}{\mathrm{vol}_{S-1}(\mathbb{S}_S)} , \tag{22}$$

where $K(A)$ is defined in equation 13, $\mathbb{S}_S$ is the unit sphere in $\mathbb{R}^S$, while $\mathrm{vol}_S(\cdot)$ means volume in S dimensions. This definition is equivalent to the one in equation 21 [10, 11].

These two equivalent definitions can be expressed in terms of an integral in the space of the growth rate vectors:

$$\Xi = \frac{2^S}{\mathrm{vol}_{S-1}(\mathbb{S}_S)} \int_{\mathbb{R}^S} \mathrm{d}^S\boldsymbol{r} \; 2\|\boldsymbol{r}\| \; \delta(\|\boldsymbol{r}\|^2 - 1) \; \prod_{i=1}^{S} \Theta(n_i^*(\boldsymbol{r})) , \tag{23}$$

where $\mathrm{vol}_{S-1}(\mathbb{S}_S)$ is the volume of the unit sphere's surface in S dimensions, $\Theta(\cdot)$ is the Heaviside function (equal to 1 is the argument is positive and to zero otherwise), and $\delta(\cdot)$ is the Dirac delta function. In this expression, we integrate over the surface of the S-dimensional unit sphere. The integral of a function $f(\boldsymbol{x})$ on the unit sphere is given by

$$\int_{\mathbb{S}_S} \mathrm{d}^S\boldsymbol{x}\, f(\boldsymbol{x}) = \int_{\mathbb{R}^S} \mathrm{d}^S\boldsymbol{x}\, 2\|\boldsymbol{x}\|\delta(\|\boldsymbol{x}\|^2 - 1)\, f(\boldsymbol{x})\,, \tag{24}$$

where the term $\delta(\|\boldsymbol{x}\|^2 - 1)$ that appears in the integration constrains $\boldsymbol{x}$ on the surface of the unit sphere, and the factor $2\|\boldsymbol{x}\|$ is the derivative of the delta function's argument, which is needed because the Dirac delta is nonlinear in $\|\boldsymbol{r}\|$. The factor $\mathrm{vol}_{S-1}(\mathbb{S}_S)$, the surface of sphere in S dimensions, can be obtained by setting $f(x) = 1$:

$$\mathrm{vol}_{S-1}(\mathbb{S}_S) = \int \mathrm{d}^S\boldsymbol{x}\, 2\|\boldsymbol{x}\|\delta(\|\boldsymbol{x}\|^2 - 1) = \frac{2\pi^{S/2}}{\Gamma(S/2)}\,, \tag{25}$$

where $\Gamma(\cdot)$ is the Gamma function. Finally, the term $\prod_{i=1}^{S} \Theta(\boldsymbol{n}_i^*(\boldsymbol{r}))$ in equation 23 expresses the constraint of all n_i^* having to be positive: this product is equal to 1 if the equilibrium $\boldsymbol{n}^*(\boldsymbol{r})$ is feasible and zero otherwise. The equilibrium $\boldsymbol{n}^*(\boldsymbol{r})$ is a function of $\boldsymbol{r}$ via equation 4.

Equation 23 defines Ξ as the volume of the domain of growth rates leading to feasible solutions. Using the results of section Supplementary Note 2, we know that if the interaction matrix $\boldsymbol{A}$ is negative definite then a feasible fixed point is globally stable. In this case Ξ is the volume of the domain of intrinsic growth rates leading to feasible and (globally) stable solutions.

Unfortunately, direct numerical computation of Ξ is inefficient when the number of species S is large. To evaluate the integral in equation 23, e.g., via Monte Carlo integration, we should draw intrinsic growth rates at random and count how many of them, out of the total, lead to a feasible equilibrium. In order to have a reliable estimate of this proportion, we should sample the space in such a way that the number of feasible growth rates found is large. This goal requires an

exponentially increasing sampling effort as S increases. In this section we provide an alternative, much faster and reliable, way of estimating Ξ.

The equilibrium solution and the growth rates are linearly related via $r_i = -\sum_{i=1}^{S} A_{ij} n_j^*$ (equation 3). Our strategy is to use this to perform a change of variables in equation 23, and integrate over $\boldsymbol{n}^*$ instead of $\boldsymbol{r}$. Since $\boldsymbol{A}$ is negative definite (and thus stable and not singular), it is invertible, and so it is always possible to perform this change of variables. Note that, more generally, the change of variables can be performed if A is nonsingular (i.e., $\det(A) = 0$). We then obtain

$$\Xi = \frac{2^S \, \Gamma(S/2) \, |\det(\boldsymbol{A})|}{2\pi^{S/2}} \int_{\mathbb{R}^S} \mathrm{d}^S \boldsymbol{n}^* \, 2\delta \left(\sum_{i,j,k} n_i^* A_{ki} A_{kj} n_j^* - 1 \right) \prod_{i=1}^{S} \Theta(n_i^*) \, , \tag{26}$$

where $|\det(\boldsymbol{A})|$ is the determinant of $\boldsymbol{A}$, which is also the Jacobian of the change of variables. After the change of variables, the integration is now performed over the feasible equilibrium points and so the condition of feasibility is automatically implemented.

It is still difficult to evaluate the previous expression numerically, because of the constraint that appears in the delta function. We can further simplify it by introducing polar coordinates. In particular, we write the vector $\boldsymbol{n}$ as $\boldsymbol{n} = n\boldsymbol{u}$, where $n = \|\boldsymbol{n}\|$ and $\boldsymbol{u}$ is a vector of unit length. We can perform a new change of variables, passing from $\boldsymbol{n}$ to n and $\boldsymbol{u}$. Specifically, for any function $f(\boldsymbol{n})$, we can write

$$\int_{\mathbb{R}^S} \mathrm{d}^S \boldsymbol{n} \, f(\boldsymbol{n}) = \int_0^\infty \mathrm{d}n \, n^{S-1} \int_{\mathbb{R}^S} \mathrm{d}^S \boldsymbol{u} \, 2\delta(\|\boldsymbol{u}\|^2 - 1) f(n\boldsymbol{u}) = \int_0^\infty \mathrm{d}n \, n^{S-1} \int_{\mathbb{S}_S} \mathrm{d}^S \boldsymbol{u} \, f(n\boldsymbol{u}) \, . \tag{27}$$

Using this expression in equation 26, we obtain

$$\Xi = \frac{2^S \, \Gamma(S/2) \, \det(\boldsymbol{A})}{2\pi^{S/2}} \int_0^\infty \mathrm{d}n \, n^{S-1} \int_{\mathbb{S}_S} \mathrm{d}^S \boldsymbol{u} \, 2\delta \left(n^2 \sum_{i,j} u_i G_{ij} u_j - 1 \right) \prod_{i=1}^{S} \Theta(u_i) \, , \tag{28}$$

where we used the fact that $\Theta(n_i) = \Theta(u_i)$ (since $n_i = nu_i$, and n is positive by definition), and we have introduced the matrix $G_{ij} = \sum_k A_{ki}A_{kj}$. We can now perform the integration over n, obtaining

$$\int_0^\infty \mathrm{d}n\, n^{S-1}\, 2\, \delta\left(n^2 \sum_{i,j} u_i G_{ij} u_j - 1\right)$$

$$= \int_0^\infty \mathrm{d}n\, n^{S-1}\, 2\, \delta\left(n - \frac{1}{\sqrt{\sum_{i,j} u_i G_{ij} u_j}}\right) \frac{1}{2n \sum_{i,j} u_i G_{ij} u_j} = \left(\sum_{i,j} u_i G_{ij} u_j\right)^{-S/2}, \tag{29}$$

and therefore the integral of equation 23 finally reads

$$\Xi = \frac{2^S\, \Gamma(S/2)\, \sqrt{\det(\boldsymbol{G})}}{2\pi^{S/2}} \int_{\mathbb{S}_S} \mathrm{d}^S \boldsymbol{u} \prod_{i=1}^{S} \Theta(u_i) \left(\sum_{i,j} u_i G_{ij} u_j\right)^{-S/2}, \tag{30}$$

where we have used the fact that $\det(\boldsymbol{G}) = \det(\boldsymbol{A}^T\boldsymbol{A}) = \det(\boldsymbol{A})^2$. In terms of the interaction matrix, the equation reads

$$\Xi = \frac{2^S\, \Gamma(S/2)\, |\det(\boldsymbol{A})|}{2\pi^{S/2}} \int_{\mathbb{S}_S} \mathrm{d}^S \boldsymbol{u} \prod_{i=1}^{S} \Theta(u_i) \left(\sum_{i,j,k} u_i A_{ki} A_{kj} u_j\right)^{-S/2}. \tag{31}$$

Equation 30 shows explicitly the role of the generators. The matrix $\boldsymbol{G}$ can indeed be rewritten as

$$G_{ik} = \sum_j g_j^i g_j^k c_i c_k = c_i c_k \boldsymbol{g}^i \cdot \boldsymbol{g}^k, \tag{32}$$

where g_j^k are the generators of the convex cone defined in equation 12 and c_i are arbitrary positive constants. Their presence, which can be seen as a change of the normalization of the vectors $\boldsymbol{g}^k$, does not affect the form of equation 30 and its dependence on $\boldsymbol{G}$ (see section Supplementary Note 3). This property can be checked explicitly from equation 30, by introducing an explicit dependence on c_i and showing that Ξ is independent of their values.

Unfortunately, the integral in equation 30 cannot be computed analytically. As mentioned before, when the integral is written in the form of equation 23 it is impractical to evaluate it numerically, since it would require an exponentially increasing sampling to get a reasonable precision. Fortunately, this is not the case when the integral is written as in equations 30 and 31. The main difference is that, after changing variables, we are directly sampling the space of feasible solutions, without losing computational time in randomly exploring the space of intrinsic growth rates looking for feasible solutions.

To evaluate the integral, we use the usual approach of Monte Carlo algorithms. In particular, it is possible to write the integral as an average over random points:

$$\frac{1}{T}\sum_{a=1}^{T}\Big(\sum_{i,j} u_i^a G_{ij} u_j^a\Big)^{-S/2} \to \frac{\Gamma(S/2)}{2\pi^{S/2}} \int d^S u \prod_{i=1}^{S} \Theta(u_i)\, 2\delta(\|u\|^2-1)\, \Big(\sum_{i,j} u_i G_{ij} u_j\Big)^{-S/2} \tag{33}$$

when $T \to \infty$. In this expression $\boldsymbol{u}^a$ are independently drawn random vectors uniformly distributed on the unit sphere and with only positive components. These two conditions are introduced to satisfy the constraints $\prod_{i=1}^{S}\Theta(u_i)$ and $2\delta(\|u\|^2-1)$ that appear in the integral. T is the sample size, and the average on the left hand side of equation 33 converges to the right hand side in the large T limit.

One always has a finite sample size T, used to approximate the integral. It is therefore important to have an estimate of the error made due to $T < \infty$. Since the left hand side of equation 33 is an average of a function over random vectors, this error can be estimated by simply using the variance of the function's values. In particular, the error σ_{MC} is defined as

$$\sigma_{\text{MC}} = \frac{1}{\sqrt{T}}\sqrt{\frac{1}{T}\sum_{a=1}^{T}\left(\sum_{i,j} u_i^a G_{ij} u_j^a\right)^{-S} - \left(\frac{1}{T}\sum_{a=1}^{T}\left(\sum_{i,j} u_i^a G_{ij} u_j^a\right)^{-S/2}\right)^2}\,. \tag{34}$$

The numerical simulation presented in the work where obtained were obtained with different

sampling effort T. Instead of fixing T a priori, we determined a precision goal, that we measured in terms of the relative error σ_{MC}/Ξ. We ran the simulations until $\sigma_{MC}/\Xi < 0.05$. In order to avoid artificially small samples and to have enough statistical power not to undershoot to much σ_{MC}, we ran $10 \times S$ Monte Carlo steps before checking the condition for the first time.

Supplementary Note 5 Stability, negative definiteness, and feasibility in random matrices

Random matrices are a useful tool in ecology, and have been studied since May's seminal paper [12]. Mostly, they have been used to model the community matrix [12, 13]. In the context of this work, we use random matrices to model interaction matrices $\boldsymbol{A}$. We consider random matrices constructed in the following way:

- $A_{ii} = -d$ where d is a positive constant.
- Each pair (A_{ij}, A_{ji}) is set equal to a pair of random variables drawn from a joint distribution with probability density function $q(x, y)$.
- The random variables are exchangeable—i.e., the probability distribution function is symmetric in its arguments: $q(x, y) = q(y, x)$—and all the moments are finite.

We show that the three most important quantities for our problem are the moments

$$E_1 = \int \mathrm{d}x\, \mathrm{d}y\; xq(x, y) = \int \mathrm{d}x\, \mathrm{d}y\; yq(x, y)\;, \tag{35}$$

$$E_2 = \sqrt{\int \mathrm{d}x\, \mathrm{d}y\; (x - E_1)^2 q(x, y)} = \sqrt{\int \mathrm{d}x\, \mathrm{d}y\; (y - E_1)^2 q(x, y)}\;, \tag{36}$$

$$E_c = \frac{1}{E_2^2} \int \mathrm{d}x\, \mathrm{d}y\; (x - E_1)(y - E_1) q(x, y)\;. \tag{37}$$

In the limit of large S, they can be computed as proper sample means of $\boldsymbol{A}$'s entries:

$$E_1 = \frac{1}{S(S-1)} \sum_{i=1}^{S} \sum_{j\neq i} A_{ij} \ , \tag{38}$$

$$E_2 = \sqrt{\frac{1}{S(S-1)} \sum_{i=1}^{S} \sum_{j\neq i} (A_{ij})^2 - E_1^2} \ , \tag{39}$$

$$E_c = \frac{1}{E_2^2} \left(\frac{1}{S(S-1)} \sum_{i=1}^{S} \sum_{j\neq i} A_{ij} A_{ji} - E_1^2 \right) \ . \tag{40}$$

The parameterization used by May [12] would correspond to

$$q_{\mathrm{May}}(x,y) = \Big((1-C)\delta(x) + Cp(x)\Big)\Big((1-C)\delta(y) + Cp(y)\Big) \ , \tag{41}$$

where $\delta(\cdot)$ is the Dirac delta function and $p(x)$ is an arbitrary distribution with mean zero and variance σ^2. The connectance C sets the probability that each entry is equal to zero (with probability $1-C$) or randomly drawn from the probability distribution $p(x)$ with probability C. In this case $E_1 = E_c = 0$, while $E_2^2 = C\sigma^2$.

In the following, we summarize known results on the spectra, negative definiteness conditions, and properties of Ξ for these matrices.

Supplementary Note 5.1 Known results on the spectra of random matrices Under the assumptions of the previous section, the eigenvalues of $\boldsymbol{A}$ in the limit of large S are uniformly distributed in an ellipse in the complex plane. If $E_1 \neq 0$ there is always an eigenvalue λ_m whose value is approximately

$$\lambda_m \approx -d + SE_1 \ , \tag{42}$$

independently of the rest of the eigenvalue distribution. The ellipse is centered at $-d - E_1$, its axes are aligned with the real and imaginary axes, and their lengths are

$$a = \sqrt{S} E_2 (1 + E_c) \tag{43}$$

and

$$b = \sqrt{S}E_2(1 - E_c)\ . \tag{44}$$

If $\lambda_m = 0$, the eigenvalue with the largest real part(s) is approximated by the rightmost point of the ellipse. The system is stable if its real part is negative. In the most general case, this condition is equivalent to

$$-d + \max\left\{SE_1, -E_1 + \sqrt{S}E_2(1 + E_c)\right\} < 0\ . \tag{45}$$

In section Supplementary Note 2 we introduced the concept of negative definiteness. In particular, we showed that when the matrix is negative definite then it is possible to disentangle stability and feasibility. The matrix is negative definite if the eigenvalues of $\boldsymbol{A} + \boldsymbol{A}^T$ are all negative. This condition reads [8]

$$-d + \max\left\{SE_1, -E_1 + \sqrt{2S(1 + E_c)}E_2\right\} < 0\ . \tag{46}$$

Figure 1 shows the values of parameters leading to the possible combinations of stability and negative definiteness in random matrices for the case $E_1 = 0$. Since we imposed that $\boldsymbol{A}$ is negative definite, the region of parameters we explore is the one above the negative definiteness line. One can see that in this way we are missing some parameterizations, corresponding to those that lead to a stable but not negative definite matrices. From equations 45 and 46 one can see that the case $E_1 < 0$ is very similar to the case $E_1 = 0$. More interestingly, for $E_1 > 0$, the conditions for stability and negative definiteness converge in the large S limit, implying that we are considering all the possible cases.

What is remarkable in these conditions and in the distribution of eigenvalues is that they are *universal* [14–17]. Universality means that they depend only on S, E_1, E_2, and E_c (and d, but via

a trivial dependence). The spectrum of eigenvalues does not depend on the detailed form of the distribution $q(x, y)$.

For instance, consider the case $q(x, y) = p(x)p(y)$, where the upper and lower triangular entries A_{ij} and A_{ji} are independent random variables. In this case $E_c = 0$ and E_1 and E_2 are the mean and standard deviation of the distribution $p(x)$. The distribution of eigenvalues and the conditions for stability and negative definiteness are the same for *any* probability distribution $p(x)$ as long as their mean E_1 and standard deviation E_2 are the same (provided some mild conditions on higher moments hold). For instance, a Lognormal distribution, a Gaussian distribution and an exponential distribution, having same mean and standard deviation, produce the same eigenvalue distribution, and therefore the same conditions for stability [18].

From an ecological perspective, one can consider different interaction matrices corresponding to different interaction types. The interaction type is given by the signs of the pairs (A_{ij}, A_{ji}): competitive interactions will have both entries with a negative sign, while in trophic interactions the entries will have opposite sign. The interaction pairs (A_{ij}, A_{ji}) for competitive interactions can for instance be obtained from the following distribution:

$$q_{\text{comp}}(x, y) = (1 - C)\delta(x)\delta(y) + Ch_-(x)h_-(y) , \tag{47}$$

where h_- is a probability distribution function with support on the negative axis (i.e., the random variables are always negative), and C is the connectance (a pair is different from zero with probability C). In the case of trophic interactions we could consider

$$q_{\text{troph}}(x, y) = (1 - C)\delta(x)\delta(y) + \frac{C}{2}p_-(x)p_+(y) + \frac{C}{2}p_+(x)p_-(y) , \tag{48}$$

where p_+ and p_- are two probability distribution functions with positive and negative support, respectively. Suppose that the moments of h_-, p_+, and p_- are chosen in such a way that $q_{\text{comp}}(x, y)$

and $q_{\text{troph}}(x, y)$ have the same values of E_1, E_2, and E_c. The interaction matrices will still look very different in the two cases: one describes a foodweb and the other a competitive system. Despite this difference, the two will have the same stability properties. In other words, different interaction types influence the stability properties of the system only via E_1, E_2 and E_c.

Supplementary Note 5.2 Universality of Ξ In this section we show that, apart from their spectral distribution, Ξ is also a universal quantity in large random matrices. That is, in the large S limit, its value does not depend on the entire distribution of the coefficients, but only on the three moments E_1, E_2, and E_c. It is important to remark that this result applies to the large S limit: the sub-leading corrections depend in principle on all the moments.

In order to show that Ξ is universal, we parameterized random networks with different distributions and checked whether Ξ depends only on E_1, E_2, E_c, and S, but not on other properties. To do this, we constructed several $S \times S$ matrices. Each individual matrix had its entries drawn from some fixed distribution, but the shape of the distribution was different across matrices. However, regardless of the distribution's shape, their moments were fixed at E_1, E_2, and E_c. We then checked whether these matrices led to the same value of Ξ.

In our simulations we considered a distribution of the pairs (A_{ij}, A_{ji}) of the form

$$q(x, y) = (1 - C)\delta(x)\delta(y) + Cp(x, y) , \tag{49}$$

where the connectance C is the probability that two species i and j interact. The probability distribution $p(x, y)$ in equation 49 depends on three parameters μ, σ, and ρ, which define the mean, variance, and correlation of the pairs drawn from $p(x, y)$. Given the values of E_1, E_2, and E_c, we can arbitrary choose C and tune μ, σ, and ρ to obtain any desired E_1, E_2, and E_c. If Ξ is universal, then different matrices built with different values of C, μ, σ, and ρ but the same values

of E_1, E_2, and E_c will lead to the same Ξ.

We considered five parameterizations of the distribution $p(x, y)$:

- Random signs, normal distribution:

$$p(x, y) = BN(x, y|\mu, \sigma, \rho) \; . \tag{50}$$

The distribution $BN(x, y|\mu, \sigma, \rho)$ is a bivariate normal distribution with marginal means equal to μ, marginal variances equal to σ^2, and correlation equal to $\rho\sigma^2$. The pairs can in principle assume all possible combinations of signs.

- Random signs, four corners:

$$\begin{aligned} p(x, y) =& \frac{q}{2}\delta(x - \mu - \sigma)\delta(y - \mu - \sigma) + \frac{q}{2}\delta(x - \mu + \sigma)\delta(y - \mu + \sigma) \\ &+ \frac{1-q}{2}\delta(x - \mu - \sigma)\delta(y - \mu + \sigma) + \frac{1-q}{2}\delta(x - \mu + \sigma)\delta(y - \mu - \sigma) \; . \end{aligned} \tag{51}$$

The pairs (x, y) can take on only four different, discrete values, potentially corresponding to all combinations on signs. The probability distribution depends on three parameters μ and σ^2 are means an variances of the distribution, while the correlation $\rho\sigma^2$ can be obtained from $\rho = 2q - 1$.

- $(+, +)$, Lognormal:

$$p(x, y) = LBN(x, y|\mu, \sigma, \rho) \; . \tag{52}$$

The distribution $LBN(x, y|\mu, \sigma, \rho)$ is a bivariate lognormal distribution with marginal means equal to $\mu > 0$, marginal variances equal to σ^2, and correlation equal to $\rho\sigma^2$. The pairs can in principle assume only positive signs. Note that not all values of ρ between -1 and 1 can be obtained when a Lognormal distribution is considered.

- $(-,-)$, Lognormal:

$$p(x, y) = LBN(-x, -y| - \mu, \sigma, \rho) \; . \tag{53}$$

This distribution takes the values drawn from a bivariate lognormal distribution, times -1. It has marginal means equal to $\mu < 0$, marginal variances equal to σ^2, and correlation equal to $\rho\sigma^2$. The pairs assume only negative signs. Note that not all values of ρ between -1 and 1 can be obtained when a Lognormal distribution is considered.

- $(+,-)$, Lognormal:

$$\begin{aligned} p(x, y) =& \frac{1}{2} LN(x|\mu_1, (1+\rho)\sigma) LN(-y| - \mu_2, (1+\rho)\sigma) \\ &+ \frac{1}{2} LN(y|\mu_1, (1+\rho)\sigma) LN(-x| - \mu_2, (1+\rho)\sigma) \; . \end{aligned} \tag{54}$$

The distribution $LN(x|\mu, \sigma)$ is Lognormal distribution with mean $\mu_1 + \mu_2$ (where $\mu_1 > 0$ and $\mu_2 < 0$), variance σ^2, and correlation $\rho\sigma^2$. The pairs assume only values with opposite signs $(+,-)$ or $(-,+)$.

In ecological terms, the first two distributions correspond to a random community (where the signs of the interaction strength are random), the $(+,+)$ case corresponds to a mutualistic community, $(-,-)$ to a competitive community, while $(+,-)$ corresponds to a food web. The mutualistic/competitive matrices can lead only to positive/negative means E_1, respectively, while the other settings can produce arbitrarily values of E_1.

Figure 2 shows the value of Ξ and of the largest eigenvalue λ for interaction matrices constructed with different connectances C and distributions, but with the same values of E_1, E_2, and E_c. As seen from the figure, the values of Ξ and λ in any particular case match up precisely with the average values over several different realizations, strongly suggesting that these two quantities are indeed universal.

Supplementary Note 6   Mean-field approximation of Ξ

The goal of this section is to compute an approximation for Ξ in the limit of large S. The volume Ξ is defined (see section Supplementary Note 4) as

$$\Xi = \frac{2^S\,\Gamma(S/2)\,\sqrt{\det(\boldsymbol{G})}}{2\pi^{S/2}} \int_{\mathbb{S}_S} \mathrm{d}^S\boldsymbol{u}\, \prod_{i=1}^{S} \Theta(u_i)\, \left(\sum_{i,j} u_i G_{ij} u_j\right)^{-S/2} , \tag{55}$$

where the matrix $\boldsymbol{G}$ can be obtained from the generators of the polytope (see equations 12 and 32), and therefore from the interaction matrix $\boldsymbol{A}$.

We can introduce a Gaussian function in equation 55 using the fact that, for any positive constant c,

$$c^{-S/2} = \frac{2}{\Gamma(S/2)} \int_0^\infty \mathrm{d}r\; r^{S-1} \exp(-cr^2)\,. \tag{56}$$

Introducing this Gaussian integral in equation 55 by letting $c = \sum_{i,j} u_i G_{ij} u_j$, we obtain

$$\Xi = \sqrt{\det(\boldsymbol{G})} \left(\frac{2}{\sqrt{\pi}}\right)^S \int_0^\infty \mathrm{d}r\; r^{S-1} \int_{\mathbb{S}_S} \mathrm{d}^S\boldsymbol{u} \left(\prod_{i=1}^{S} \Theta(u_i)\right) \exp\left(-r^2 \sum_{i,j} u_i G_{ij} u_j\right) , \tag{57}$$

which can be rewritten as

$$\Xi = \sqrt{\det(\boldsymbol{G})}\Big(\frac{2}{\sqrt{\pi}}\Big)^S \int_{\mathbb{R}^S} \mathrm{d}^S\boldsymbol{z}\, \Big(\prod_{i=1}^{S} \Theta(z_i)\Big)\, \exp\Big(-\sum_{i,j} z_i G_{ij} z_j\Big)\,, \tag{58}$$

where $z_i = r u_i$. We can rewrite this equation as

$$\Xi = \sqrt{\det(\boldsymbol{G})} \left(\frac{2}{\sqrt{\pi}}\right)^S \int_{\mathbb{R}^S} \mathrm{d}^S\boldsymbol{z}\, \prod_{i=1}^{S} \left(\Theta(z_i)\, e^{-z_i^2} \exp\left(-\sum_{j\neq i} z_i G_{ij} z_j\right)\right) , \tag{59}$$

where we used the fact that the diagonal entries of $\boldsymbol{G}$, when expressed in terms of the normalized generators, are equal to one.

The reader familiar with statistical mechanics will notice that equation 59, which can be written as

$$\Xi \propto \int_{\mathbb{R}^S} \mathrm{d}^S\boldsymbol{z}\; q(\boldsymbol{z})\, \prod_{i=1}^{S} \left(\exp\left(-\sum_{j\neq i} z_i G_{ij} z_j\right)\right) , \tag{60}$$

has the form of a partition function. For instance one can recover the Ising model [19] with the choice $q(\boldsymbol{z}) = \prod_i \delta(z_i^2 = 1)$ or the spherical model [20] when $q(\boldsymbol{z}) = \delta(S - \sum_i z_i^2)$. The term $z_i G_{ij} z_j$ in particular plays the role of the interactions of the system.

Integrals of the form 60 are the most studied objects of statistical mechanics, and yet in most cases are not analytically solvable. There are, on the other hand, many techniques that can be used to obtain good approximations to 60. The most celebrated one is probably the mean-field approximation [19] and it is the one we are using in this section. In particular, the idea of the mean-field approximation is to replace the interactions of an entity (spins in the case of the Ising model or species in our case) with an average "effective" interaction. This reduces a many-body problem, where all interactions of spins or populations are coupled, into an effective one-body problem.

If the system is large enough (in our case if $S \to \infty$), the mean-field approximation is know to be exact in the case of "fully connected" interactions. In terms of equation 60, this corresponds to a matrix $\boldsymbol{G}$ with the same constant in all its offdiagonal entries. The matrix $\boldsymbol{G}$ is constant when $\boldsymbol{A}$ has constant offdiagonal entries. We will consider therefore the case of $\boldsymbol{A}$'s diagonal entries being equal to -1 and its offdiagonal entries to a constant E_1. Using equation 12, the ith component of the kth generator is then

$$g_i^k = -\frac{E_1}{1 + (S-1)E_1^2} \tag{61}$$

for $i \neq k$, and

$$g_k^k = \frac{1}{1 + (S-1)E_1^2}\ . \tag{62}$$

Using equation 32, we therefore obtain that the diagonal entries of $\boldsymbol{G}$ are equal to 1, while the

offdiagonal ones are constant and equal to

$$G_{ij} = \frac{-2E_1 + (S-2)E_1^2}{1 + (S-1)E_1^2} \ . \tag{63}$$

We define the constant β as

$$\beta = S\frac{-2E_1 + (S-2)E_1^2}{1 + (S-1)E_1^2} \ , \tag{64}$$

and therefore we have $G_{ii} = 1$ and $G_{ij} = \beta/S$ for $i \neq j$. The determinant of $\boldsymbol{G}$ in this case turns out to be

$$\det(\boldsymbol{G}) = \left(1 + \frac{S-1}{S}\beta\right)\left(1 - \frac{\beta}{S}\right)^{S-1} \approx (1+\beta)e^{-\beta} \ , \tag{65}$$

where the last form holds for large S. In this case of constant interactions, we obtain, from equation 59,

$$\begin{aligned} \Xi =& \sqrt{\det(\boldsymbol{G})}\left(\frac{2}{\sqrt{\pi}}\right)^S \int_{\mathbb{R}^S} \mathrm{d}^S\boldsymbol{z} \prod_{i=1}^{S}\left(\Theta(z_i)\, e^{-z_i^2} \exp\left(-z_i\frac{\beta}{S}\sum_{j\neq i} z_j\right)\right) = \\ =& \sqrt{\det(\boldsymbol{G})}\left(\frac{2}{\sqrt{\pi}}\right)^S \int_{\mathbb{R}^S} \mathrm{d}^S\boldsymbol{z} \left(\prod_{i=1}^{S}\Theta(z_i)\right) \exp\left(-\sum_i z_i^2 - \frac{\beta}{S}(\sum_i z_i)^2\right) \ , \end{aligned} \tag{66}$$

up to subleading terms in S.

Equation 66 can be written as

$$\Xi = \sqrt{\det(\boldsymbol{G})}\left(\frac{2}{\sqrt{\pi}}\right)^S Z_h \left\langle \exp\left(-\frac{\beta}{S}(\sum_i z_i)^2 + h\sum_i z_i\right)\right\rangle_h \ , \tag{67}$$

where

$$\begin{aligned} Z_h :=& \int_{\mathbb{R}^S} \mathrm{d}^S\boldsymbol{z} \left(\prod_{i=1}^{S}\Theta(z_i)\right) \exp\left(-\sum_i z_i^2 - h\sum_i z_i\right) = \\ =& \left(\int_0^\infty \mathrm{d}z\, e^{-z^2 - hz}\right)^S = \left(\frac{\sqrt{\pi}}{2}e^{h^2/4}\operatorname{erfc}(h/2)\right)^S \ , \end{aligned} \tag{68}$$

where $\operatorname{erfc}(\cdot)$ is the complementary error function, defined as

$$\operatorname{erfc}(x) = \frac{2}{\sqrt{\pi}}\int_x^\infty \mathrm{d}t\, e^{-t^2} \ . \tag{69}$$

The average $\langle\cdot\rangle_h$ is defined as

$$\Big\langle f(\boldsymbol{z})\Big\rangle_h := \frac{1}{Z_h}\int_{\mathbb{R}^S} \mathrm{d}^S\boldsymbol{z}\ \left(\prod_{i=1}^S \Theta(z_i)\right)\ \exp\left(-\sum_i z_i^2 - h\sum_i z_i\right) f(\boldsymbol{z})\ . \tag{70}$$

Using Jensen's inequality in equation 70 we have that

$$\begin{aligned}\Xi =&\sqrt{\det(\boldsymbol{G})}\left(\frac{2}{\sqrt{\pi}}\right)^S Z_h\Big\langle\exp\left(-\frac{\beta}{S}(\sum_i z_i)^2 + h\sum_i z_i\right)\Big\rangle_h \geq \\ &\geq \sqrt{\det(\boldsymbol{G})}\left(\frac{2}{\sqrt{\pi}}\right)^S Z_h \exp\left(\Big\langle -\frac{\beta}{S}(\sum_i z_i)^2 + h\sum_i z_i\Big\rangle_h\right)\ .\end{aligned} \tag{71}$$

In the following we will approximate the first expression with the second one. It is possible to prove that, in the large S limit, the second expression converges to the first one.

Applying the mean-field approximation we neglect fluctuations of the variables, i.e. we have

$$\Big\langle -\frac{\beta}{S}(\sum_i z_i)^2 + h\sum_i z_i\Big\rangle_h = -\frac{\beta}{S}\Big\langle(\sum_i z_i)^2\Big\rangle_h + h\sum_i\langle z_i\rangle_h \approx S\left(-\beta m^2 + hm\right)\ , \tag{72}$$

where

$$m := \langle z_i\rangle_h = -\frac{1}{S}\frac{\partial}{\partial h}\log(Z_h)\ . \tag{73}$$

By introducing equation 72 in equation 71 we have

$$\Xi \approx \sqrt{\det(\boldsymbol{G})}Z_h\left(\frac{2}{\sqrt{\pi}}\exp\left(-\beta m^2 + hm\right)\right)^S = \Xi_{MF}\ . \tag{74}$$

This equation is a function of h, which is a free parameter. Since it is a lower bound for the actual value of Ξ, the best approximation would correspond to the value of h which maximizes the approximation. We have therefore that h is a solution of the following equation

$$0 = \frac{\partial}{\partial h}\log(\Xi_{MF}) = \frac{\partial}{\partial h}\log(Z_h) + S\frac{\partial}{\partial h}(-\beta m^2 + hm) = S(h - 2\beta m)\frac{\partial m}{\partial h}\ , \tag{75}$$

where m is given by equation 73. We obtain therefore $m = h/(2\beta)$ and then, by neglecting sub-leading terms in S and introducing $m = h/(2\beta)$ in equation 74

$$\frac{1}{S}\log\Xi_{MF} \approx \log\left(\mathrm{erfc}(h/2)\exp\left(\frac{h^2}{4}\frac{1+\beta}{\beta}\right)\right)\ . \tag{76}$$

By maximizing this equation respect to h we obtain

$$0 = \frac{\partial}{\partial h} \log(\Xi_{MF}) = \frac{h}{2}\left(\frac{1}{\beta}+1\right) + \frac{\partial}{\partial h}\log\left(\mathrm{erfc}(h/2)\right) = \frac{h}{2}\left(\frac{1}{\beta}+1\right) - \frac{e^{-h^2/4}}{\sqrt{\pi}\,\mathrm{erfc}(h/2)} \ . \quad (77)$$

Equation 77 cannot be solved exactly. By expanding around $h = 0$ we obtain

$$0 = \frac{h}{2}\left(\frac{1}{\beta}+1\right) - \frac{1}{\sqrt{\pi}} - \frac{h}{\pi} \ , \quad (78)$$

which is solved by

$$h = \frac{2\beta\sqrt{\pi}}{\pi + \beta(\pi - 2)} \ . \quad (79)$$

One can observe that the solution $h = 0$ corresponds to $\beta = 0$, i.e. to a non-interacting ecosystem. Expanding around $h = 0$ is therefore meaningful when the interactions are not too strong. It is possible to verify that the approximate solution 79 is very close to the actual solution obtained by solving numerically equation 77 also for not too small values of β

Using equation 79 into equation 76 we obtain

$$\frac{1}{S}\log\Xi_{MF} \approx \frac{\beta(1+\beta)\pi}{(\pi + \beta(\pi-2))^2} + \log\mathrm{erfc}\left(\frac{\sqrt{\pi}\beta}{\pi + \beta(\pi - 2)}\right) \ , \quad (80)$$

which is our final result. In figure 3 we compare this equation with the volume computed numerically in the case of constant interactions, finding a very good match.

In the most general case of an interaction matrix with nonconstant offdiagonal entries, we can consider equation 72 as an approximation valid in the case of $E_2 \to 0$. As β was defined in terms of the generators, we can extend the approximation to the case $E_2 > 0$ by considering β as the expected value of $\boldsymbol{G}$'s entries, which corresponds to the average overlap of two rows of the interaction matrix $\langle\cos(\eta)\rangle$, defined in equation 109. In this more general case the mean-field value

of Ξ is expected to be a good approximation when $\mathrm{var}(\cos(\eta))$ is small enough. By substituting $\beta = \langle\cos(\eta)\rangle$, using equation 112, into equation 72 we obtain

$$\begin{aligned}\frac{1}{S}\log(\Xi) \approx & \frac{\pi E_1(2d - E_1 S)\left(2dE_1 + d - S\left(2E_1^2 + E_2^2\right)\right)}{\left(d(2(\pi-2)E_1+\pi) - S\left(2(\pi-1)E_1^2 + \pi E_2^2\right)\right)^2} \\ & \log\left(\mathrm{erfc}\left(\frac{\sqrt{\pi}E_1(E_1 S - 2d)}{S\left(2(\pi-1)E_1^2+\pi E_2^2\right) - d(2(\pi-2)E_1+\pi)}\right)\right) \, .\end{aligned} \tag{81}$$

When $\mathrm{var}(\cos(\eta))$ is not small, we observed that the empirical formula

$$\begin{aligned}\frac{1}{S}\log(\Xi) \approx & \frac{\pi E_1(2d - E_1 S)\left(2dE_1 + d - S\left(2E_1^2 + E_2^2\right)\right)}{\left(d(2(\pi-2)E_1+\pi) - S\left(2(\pi-1)E_1^2 + \pi E_2^2\right)\right)^2} \\ & \log\left(\mathrm{erfc}\left(\frac{\sqrt{\pi}E_1(E_1 S - 2d)}{S\left(2(\pi-1)E_1^2+\pi E_2^2\right) - d(2(\pi-2)E_1+\pi)}\right)\right) + \\ & + \log\left(1 + \frac{3SE_2^2(1+E_c)}{2\pi}\right) \, .\end{aligned} \tag{82}$$

explains well the values obtained in simulations. This is the formula we used to make figure 2 in the main text.

In order to simplify the expression and make it more readable, we can expand equation 80 around $\beta = 0$, i.e., when the interactions between species are small. By expanding $(\Xi_{MF})^{1/S}$ around $\beta = 0$ and taking the logarithm of the expression, we obtain

$$\frac{1}{S}\log \Xi_{MF} \approx \log\left(1 - \frac{\beta}{\pi}\right) \, . \tag{83}$$

Equation 2 of the main text was obtained by substituting $\beta = \langle\cos(\eta)\rangle$, using equation 112, in the case of $E_2 = 0$.

Supplementary Note 7 Feasibility of consumer-resource communities

This section considers explicitly a community with two trophic levels and consumer-resource interactions. While empirical communities have a more complicated interaction structure, this example is particularly relevant to better understand how Ξ should be interpreted.

We consider a system with S_R resource and S_C consumer ($S_R + S_C = S$) populations, whose dynamics is described by equation 1 with the interaction matrix

$$\boldsymbol{A} = \begin{pmatrix} -\boldsymbol{C} & -\boldsymbol{B} \\ \boldsymbol{Z}\boldsymbol{B}^T\boldsymbol{W} & \boldsymbol{0} \end{pmatrix} , \tag{84}$$

where $\boldsymbol{C}$ is an $S_R \times S_R$ nonnegative matrix, $\boldsymbol{B}$ is an $S_R \times S_C$ nonnegative matrix, while $\boldsymbol{Z}$ and $\boldsymbol{W}$ are two positive diagonal matrices of dimension $S_C \times S_C$ and $S_R \times S_R$, respectively.

If $\boldsymbol{C}$ is a positive diagonal matrix, any feasible fixed point is globally asymptotically stable [21]. When $\boldsymbol{C}$ is not diagonal, one can prove that any feasible fixed point is globally asymptotically stable if $\boldsymbol{C}\boldsymbol{W}^{-1}$ is positive definite (i.e., $-\boldsymbol{C}\boldsymbol{W}^{-1}$ is negative definite). Assuming that this condition holds, stability of feasible fixed points is ensured and we can study feasibility alone.

Using equation 3, we obtain the equations

$$r_i^R = \sum_{j=1}^{S_R} C_{ij} n_j^{R*} + \sum_{j=1}^{S_C} B_{ij} n_j^{C*} , \tag{85}$$

$$-r_i^C = \sum_{j=1}^{S_R} Z_i B_{ji} W_j n_j^{R*} , \tag{86}$$

where $\boldsymbol{r}^R$ and $\boldsymbol{r}^C$ are the intrinsic growth rates of resources and consumers, while $\boldsymbol{n}^{R*}$ and $\boldsymbol{n}^{C*}$ are their equilibrium abundances. Since all the matrices that appear in this equation are nonnegative, an intrinsic growth rate vector is contained in the feasibility domain only if $r_i^R > 0$ for all $i = 1, \ldots, S_R$ and $r_i^C < 0$ for all $i = 1, \ldots, S_C$. An intrinsic growth rate vector that does not respect these conditions is not in the feasibility domain. The feasibility domain is therefore fully contained in one orthant, implying that the maximum value of its size is $\Xi = 1$.

The S-dimensional volume of the feasibility domain is nonzero only if it is defined by S linearly independent generators. The generators of the feasibility domain are proportional to the

columns of the interaction matrix. If the interaction matrix has the form of equation 84, S_R generators will have the form $\boldsymbol{g} = (\boldsymbol{v}, \boldsymbol{0})$, where $\boldsymbol{v}$ has S_C components. These generators can be linearly independent only if $S_R \geq S_C$, and therefore $\Xi > 0$ only if $S_R \geq S_C$. More generally, if $\det(A) = 0$, then $\Xi = 0$ [22].

Assuming that the determinant of A is different from zero, we can use equation 26 obtaining

$$\Xi = \sqrt{\det(\boldsymbol{A})} \left(\frac{2}{\sqrt{\pi}}\right)^S \int_{\mathbb{R}^S} \mathrm{d}^S \boldsymbol{z} \left(\prod_{i=1}^{S} \Theta(z_i)\right) \exp\left(\sum_{ij} z_i A_{ij} z_j\right) . \tag{87}$$

Given the structure of the matrix A, it is convenient to write $\boldsymbol{z} = (\boldsymbol{v}, \boldsymbol{u})$, where $\boldsymbol{v}$ and $\boldsymbol{u}$ are two vectors with S_R and S_C components respectively. The argument of the exponential can be rewritten as

$$\sum_{ij} z_i A_{ij} z_j = -\sum_{i=1}^{S_R}\sum_{j=1}^{S_R} v_i C_{ij} v_j - \sum_{i=1}^{S_R}\sum_{j=1}^{S_C} v_i B_{ij}(1 - Z_i W_j) u_j . \tag{88}$$

By integrating over the variables $\boldsymbol{u}$, we finally obtain

$$\Xi = \sqrt{\det(\boldsymbol{A})} \left(\frac{2}{\sqrt{\pi}}\right)^S \int_{\mathbb{R}^S} \mathrm{d}^{S_R} \boldsymbol{v} \left(\prod_{i=1}^{S_R} \Theta(v_i)\right) \exp\left(\sum_{ij} v_i C_{ij} v_j\right) \frac{1}{\prod_{j=1}^{S_C} \sum_{i=1}^{S_R} v_i B_{ij}(1 - Z_i W_j)} . \tag{89}$$

Figure 4 shows the size of the feasibility domain of a consumer-resource community, computed using Monte Carlo integration as explained in section Supplementary Note 4. We consider an interaction matrix with the structure of equation 84, with a diagonal $\boldsymbol{C}$ (i.e., $C_{ij} = 1$ if $i = j$ and zero otherwise) and scalar matrices $\boldsymbol{Z}$ and $\boldsymbol{W}$ (i.e., $Z_{ii} = W_{ii} = \sqrt{\eta}$ and $Z_{ij} = W_{ij} = 0$ if $i \neq j$). The elements of the rectangular matrix $\boldsymbol{B}$ were independently drawn from a lognormal distribution with mean μ and variance $c_v^2 \mu^2$, where c_v is the coefficient of variation. Since $\boldsymbol{C}$ is equal to the identity matrix, then the interaction matrix is diagonally stable and therefore any feasible point is globally stable [21]. Figure 4 shows the effect of η, μ and c_v on the size Ξ of the feasibility domain. Interestingly, η and μ have a small effect on Ξ, while the coefficient of variation

has a strong influence on it. It is important to notice that, as explained above, as the interspecific interaction goes to zero (and therefore both c_v and μ tend to zero), $\Xi \to 0$ as well.

Supplementary Note 8 Empirical networks and randomizations

We considered 89 mutualistic networks and 15 food webs. Empirical networks are encoded in terms of adjacency matrices $\boldsymbol{L}$, with $L_{ij} = 1$ if species j affects species i and zero otherwise.

Supplementary Note 8.1 Mutualistic networks The 89 mutualistic networks (59 pollination networks and 30 seed-dispersal networks) were obtained from the Web of Life dataset (`www.web-of-life.es`) where references to the original works can be found. When the original network was not fully connected, we considered the largest connected component.

In the case of mutualistic networks, the adjacency matrix $\boldsymbol{L}$ is bipartite, i.e., it has the structure

$$\boldsymbol{L} = \begin{pmatrix} 0 & \boldsymbol{L}_b \\ \boldsymbol{L}_b^T & 0 \end{pmatrix} , \tag{90}$$

where $\boldsymbol{L}_b$ is a $S_A \times S_P$ matrix (S_A and S_P being the number of animals and plants respectively). The adjacency matrix contains information only about the interactions between animals and plants, but not about competition within plants or animals.

We parameterized the interaction matrix in the following way:

$$\boldsymbol{A} = \begin{pmatrix} \boldsymbol{W}^A & \boldsymbol{L}_b \circ \boldsymbol{W}^{AP} \\ \boldsymbol{L}_b^T \circ \boldsymbol{W}^{PA} & \boldsymbol{W}^P \end{pmatrix} , \tag{91}$$

where the symbol $\circ$ indicates the Hadamard or entrywise product (i.e., $(\boldsymbol{A} \circ \boldsymbol{B})_{ij} = A_{ij}B_{ij}$), while $\boldsymbol{W}^A$, $\boldsymbol{W}^{AP}$, $\boldsymbol{W}^{PA}$, and $\boldsymbol{W}^P$ are all random matrices. $\boldsymbol{W}^A$ and $\boldsymbol{W}^P$ are both square matrices (of

dimension $S_A \times S_A$ and $S_P \times S_P$), while $\boldsymbol{W}^{AP}$ and $\boldsymbol{W}^{PA}$ are rectangular matrices of size $S_A \times S_P$ and $S_P \times S_A$ respectively. The diagonal elements W^A_{ii} and W^P_{ii} were set to -1, while the pairs (W^A_{ij}, W^A_{ji}) and (W^P_{ij}, W^P_{ji}) were drawn from a bivariate normal distribution with mean μ_-, variance $\sigma_+^2 = c\mu_-^2$, and correlation $\rho\sigma_+^2$. Since these two matrices represent competitive interactions, $\mu_- < 0$. The the pairs $(W^{AP}_{ij}, W^{PA}_{ji})$ were extracted from a bivariate normal distribution with mean μ_+, variance $\sigma_-^2 = c\mu_+^2$, and correlation $\rho\sigma_-^2$, where $\mu_+ > 0$.

We analyze more than 600 parameterizations, obtained by considering different values of μ_-, μ_+, c, and ρ. For each network and parametrization we computed the size of feasibility domain Ξ. The bottom panel of Figure 2 in the main text was obtained by comparing Ξ obtained in this way with the analytical prediction obtained in equation 81.

Supplementary Note 8.2 Food webs A summary of the properties and reference of the food webs can be found in table 1. In the case of food webs the adjacency matrix L is not symmetric, and an entry $L_{ij} = 1$ indicates that species j consumes species i. We removed all cannibalistic loops. Since both L_{ij} and L_{ji} are never simultaneously equal to one (there are no loops of length two), we parameterized the offdiagonal entries of $\boldsymbol{A}$ as

$$A_{ij} = W^+_{ij} L_{ij} + W^-_{ji} L_{ji} \ , \tag{92}$$

while the diagonal was fixed at -1. Both $\boldsymbol{W}^+$ and $\boldsymbol{W}^-$ are random matrices, where the pairs (W^+_{ij}, W^-_{ij}) are drawn from a bivariate normal distribution with marginal means (μ_+, μ_-) and correlation matrix

$$\begin{pmatrix} c\mu_+^2 & \rho c\mu_+^2 \\ \rho c\mu_-^2 & c\mu_-^2 \end{pmatrix} \tag{93}$$

We analyzed more than 200 parameterizations, obtained by considering different values of

μ_-, μ_+, c, and ρ. For each network and parametrization we computed the size of feasibility domain Ξ. The bottom panel of Figure 2 in the main text was obtained by comparing Ξ obtained in this way with the analytical prediction obtained in equation 81. In this case the analytical prediction overestimate the actual value of Ξ, indicating that there is a role of structure in determining structural stability.

Supplementary Note 9 Randomization of empirical networks: assessing the role of structure

Supplementary Note 9.1 Mutualistic networks We compared the size of the feasibility domain obtained for empirical networks with the corresponding randomizations. For each network we randomized the block $\boldsymbol{L}_b$ 100 times, by generating connected networks with same size and number of links. We parameterized each randomized network independently as described in section Supplementary Note 8, and we compared their properties with those of the empirical network, parameterized independently 100 times. Figure 5 shows the comparison between Ξ of random and empirical networks. As expected from the fact that the analytical prediction for random matrices works well, the empirical values and the values obtained with randomizations are compatible. Comparing this figure with figure 2 of the main text we observe that the empirical values and the ones obtained with randomizations match also in the cases were the analytical approximation failed. This implies that the reason of the mismatch is due to the difference between the analytical approximation and the randomizations, and it is not due to the specific structure of the empirical interactions. There are two main sources of errors in this case. On one hand, ours analytical prediction is expected to work is the number of species is large enough and if the variance of interactions is not to high (that is not always true for the parametrizations used). On the other hand, our approximation was formulated for random matrices, while randomizations of mutualistic networks

still conserve a bipartite structure.

The randomization procedure explained above and figure 5 show that the size of the coexistence domain obtained with empirical network structure is well predicted by the one obtained with random structure. This result does not imply that structure has no effect on Ξ, but it shows that, if this effect exists, it must be relatively small (compared for instance to the variation of Ξ obtained by changing the interaction strengths), i.e. the relative error made by approximating empirical networks with random structure must be small.

Since the effect of structure is small, it is also expected to be very sensible to the interaction strengths. When we parametrized empirical networks and their randomizations to obtain figure 5, we drawn the interaction strengths several times from a given distributions. The realized coefficients were therefore different across different networks, and the values of Ξ shown in figure 5 were averaged over these independent extractions. Since the difference between randomizations and empirical structure is small, it might be impossible to detect any difference with this procedure.

In order to explore and quantify the effect of the empirical structure on the size of feasibility domain, we adopted a different parametrization and randomization method. Given an empirical network, we drawn the interaction strengths only once from a given distribution (as described in section Supplementary Note 8). Using this list of interaction strenghts we parametrized 100 times each empirical network. Different parametrization differ in the position of the coefficients, but not in their values that are conserved across parametrizations. We then compared their size of feasibility domain with the one obtained by parameterizing with the same list of coefficients 100 randomized networks obtained as explained above.

Figures 6, 7, 8 and 9 show the results obtained for different distributions of interaction

strengths (parametrized as explained in section Supplementary Note 8). In absence of competition and in absence of variation in the interaction strengths, there is the maximum observable effect. As the competition level is increased and once variation in the interaction strengths is introduced, the effect of the network topology on the total size of feasibility domain becomes negligible.

Supplementary Note 9.2 Food webs We compared the size of feasibility domain of empirical networks with their corresponding randomizations and a network generated accordingly to the cascade model [23].

For each network, we randomized the adjacency matrix $\boldsymbol{L}$ 100 times, by generating connected networks with the same size and number of links.

We also generated networks generated accordingly to the cascade model (using the same method explained in [24]). In this case the adjacency matrix was obtained by generating connected networks with the same size and number of links, by assigning a link between species i and j only if $i > j$.

Figure 10 is the same as figure 2 of the main text, with the addition of randomizations and networks generated with the cascade model. As expected the analytical prediction works very well in describing random networks, while it fails significantly to predict the size of the feasibility domain of cascade and empirical networks. To better quantity the difference between those empirical structures and randomizations, we compared each network separately in figure 11 and 12. We observe that random networks have always larger feasibility domain than networks generated by the cascade model and the empirical ones. Networks generated via the cascade model almost always overestimate the empirical feasibility domains, showing that empirical network structure has a significant negative effect on the size of the feasibility domain.

Supplementary Note 10 Distribution of side lengths

In section Supplementary Note 3 we showed that the feasibility domain is a convex polyhedral cone in the space of intrinsic growth rates $\boldsymbol{r}$. Since the stationary solution of equation 1 is linear in $\boldsymbol{r}$, we can study the feasibility domain considering only vectors on the unit sphere's surface. In section Supplementary Note 4 we defined Ξ, which quantifies the volume of the feasibility domain.

The size of the feasibility domain, i.e., how many combinations of the intrinsic growth rates correspond to a feasible fixed point, is not the only interesting property. Two systems having the same number of feasible combinations of growth rates (i.e., the same value of Ξ), can respond very differently to perturbations of the growth rates. We imagine here that a perturbation (e.g., a change of the abiotic conditions) correspond to a change in the growth rate vector. Since we can consider normalized growth rate vectors (because of the linearity of the equations), the effect of a perturbation on feasibility depends only on the angular change of the growth rate vector and not on its length.

The volume Ξ quantifies how many growth rate vectors are compatible with coexistence. Let us consider a feasible growth rate vector, and perturb it in a random direction. What is the probability that the new vector is still feasible? This is not just a function of the size Ξ of the feasibility domain. Indeed, one can imagine that the feasibility domain is about equally spread in every direction—or that, for the exact same value of Ξ, the feasibility domain is streched in some directions but is very narrow in some other ones. A perturbation in one of the "narrow" directions is much more likely to lead out of the feasibility domain in the latter case than in the former.

To quantify this property, one strategy could be to measure the different responses on the perturbation (i.e., the probability of being feasible) depending on the direction of the perturbation

(in which direction we change the growth rate vector). This choice has the big disadvantage of depending not only on the properties of interactions (the interaction matrix $\boldsymbol{A}$), but also on the strength of the perturbation (the angular displacement between the initial and the final growth rate vector) and the growth rate vector before the perturbation (e.g., if the initial vector is close or far from the edge of the feasibility domain). We propose instead a purely geometrical method to quantify the response to different perturbations (see figure 1 of the main text).

The feasibility domain, when restricted to the surface of a hypersphere, can be imagined as the generalization of a triangle on a sphere (see section Supplementary Note 12). The natural, geometric quantities bounding the maximal perturbation that will leave the system feasible, are the lengths of the triangle's sides. When S species are considered, there are $S(S-1)/2$ sides. Their lengths measure the maximum permissible perturbation of the growth rates in the corresponding direction if one is to retain feasibility. This property has the advantage of being purely geometrical, depending only on the interactions (via the interaction matrix) and not, for instance, on any choice of the initial conditions.

We can measure the distribution of the side lengths. Imagine we have two interaction matrices with the same Ξ, but with very different distributions of side lengths. One of them has all sides of equal length, while the other one has a more heterogeneous distribution. In the first case any direction of the perturbation is expected to have a similar effect, and there are no particularly dangerous directions. In the second case there are some directions of the perturbation that are much more dangerous than others, and even a small change of conditions along one of those dangerous direction can lead to the extinction of one or more species.

We know that the feasibility domain is a convex polyhedral cone (see section Supplementary

Note 3). Its "corners" are identified by its generators and its sides are determined by all pairs of generators (see section Supplementary Note 12 for the $S = 3$ case).

Since we are considering growth rates on the unit (hyper)sphere, and the generators are normalized to one, any pair of generators will lie on the sphere's surface. The scalar product of two generators is the cosine of the angle between the two. Since the two generators are on the unit ball's surface, the arc between the two (which is the side length) is equal to the angle. We have therefore that the length of the side of the feasibility domain corresponding to a pair of generators $\boldsymbol{g}^i$ and $\boldsymbol{g}^j$ is

$$\eta_{ij} = \arccos\left(\boldsymbol{g}^i \cdot \boldsymbol{g}^j\right) \ . \tag{94}$$

Using equation 12, we can express the $S(S-1)/2$ side lengths of the convex polytope explicitly in terms of the interaction matrix:

$$\eta_{ij} = \arccos\left(\frac{\sum_k A_{ki}A_{kj}}{\sqrt{\sum_k A_{ki}A_{ki}\sum_l A_{lj}A_{lj}}}\right) \ . \tag{95}$$

We are interested in the distribution of the side lengths, and in particular in its heterogeneity. In the following section we will calculate these quantities for random matrices.

Supplementary Note 10.1 The distribution of side lengths in random matrices In this section we obtain the distribution of sides length for large random matrices, whose entries are distributed accordingly to an arbitrarily bivariate distribution.

We assume that the diagonal elements of $\boldsymbol{A}$ are all equal to $-d$ (this hypothesis can be easily generalized), while the offdiagonal pairs (A_{ij}, A_{ji}) are random variables with distribution $q(x, y)$.

Our goal is to find the distribution of the side lengths η in the large S limit, defined as

$$P(\eta) = \lim_{S\to\infty} \frac{1}{S(S-1)} \sum_{i\neq j} \int \prod_{m>n} \Big(\mathrm{d}A_{mn} \mathrm{d}A_{nm} q(A_{mn}, A_{nm}) \Big) \\ \times \delta \left(\eta - \arccos\left(\frac{\sum_k A_{ki} A_{kj}}{\sqrt{\sum_k A_{ki} A_{ki} \sum_l A_{lj} A_{lj}}} \right) \right), \tag{96}$$

Since we are summing over all i and j, and all the rows are identically distributed, we can remove the sum and consider just two rows:

$$P(\eta) = \lim_{S\to\infty} \int \prod_{m>n} \Big(\mathrm{d}A_{mn} \mathrm{d}A_{nm} q(A_{mn}, A_{nm}) \Big) \\ \times \delta \left(\eta - \arccos\left(\frac{\sum_k A_{k1} A_{k2}}{\sqrt{\sum_k A_{k1} A_{k1} \sum_l A_{l2} A_{l2}}} \right) \right), \tag{97}$$

Since we are interested in the large S limit, we have that

$$\begin{aligned} \sum_k A_{k1} A_{k1} =& A_{11} + \sum_{k>1} (A_{k1})^2 \approx -d + (S-1) \int \mathrm{d}x \mathrm{d}y \; q(x,y) \; x^2 \\ =& -d + (S-1)(E_1^2 + E_2^2), \end{aligned} \tag{98}$$

where E_1 and E_2 are the first and second marginal moments of q (equations 35 and 36). Let us call

this quantity Z. In this limit we therefore obtain

$$\begin{aligned}
P(\eta) &= \lim_{S\to\infty} \int \prod_{m>n} \Big(\mathrm{d}A_{mn}\mathrm{d}A_{nm}q(A_{mn},A_{nm})\Big)\ \delta\left(\eta - \arccos\left(\frac{\sum_k A_{k1}A_{k2}}{Z}\right)\right) \\
&= \lim_{S\to\infty} \int \prod_{m>n} \Big(\mathrm{d}A_{mn}\mathrm{d}A_{nm}q(A_{mn},A_{nm})\Big)\ Z\,|\sin(\eta)|\ \delta\left(Z\cos(\eta) - \sum_k A_{k1}A_{k2}\right) \\
&= Z|\sin(\eta)| \lim_{S\to\infty} \int \prod_{m>n} \Big(\mathrm{d}A_{mn}\mathrm{d}A_{nm}q(A_{mn},A_{nm})\Big)\ \delta\left(Z\cos(\eta) - \sum_k A_{k1}A_{k2}\right) \\
&= Z|\sin(\eta)| \lim_{S\to\infty} \int \prod_{m>n} \Big(\mathrm{d}A_{mn}\mathrm{d}A_{nm}q(A_{mn},A_{nm})\Big) \\
&\quad \times \delta\left(Z\cos(\eta) - A_{11}A_{21} - A_{22}A_{12} - \sum_{k>2} A_{k1}A_{k2}\right) \\
&= Z|\sin(\eta)| \lim_{S\to\infty} \int \prod_{m>n} \Big(\mathrm{d}A_{mn}\mathrm{d}A_{nm}q(A_{mn},A_{nm})\Big) \\
&\quad \times \delta\left(Z\cos(\eta) + d(A_{12}+A_{21}) - \sum_{k>2} A_{k1}A_{k2}\right) \\
&= Z|\sin(\eta)| \int \mathrm{d}t \int \mathrm{d}s \int \mathrm{d}A_{12}\mathrm{d}A_{21}q(A_{12},A_{21})\delta(t - A_{12} - A_{21}) \\
&\quad \times \int \prod_{k>2} \mathrm{d}A_{k1}\mathrm{d}A_{k2}q(A_{k1})q(A_{k2})\delta\left(s - \sum_{k>2} A_{k1}A_{k2}\right) \\
&\quad \times \delta\left(Z\cos(\eta) + dt - \sum_{k>2} A_{k1}A_{k2}\right) \\
&= Z|\sin(\eta)| \int \mathrm{d}t \int \mathrm{d}s \int \mathrm{d}x\mathrm{d}y\ q(x,y)\delta(t-(x+y)) \\
&\quad \times \int \left(\prod_{k=1}^{S-2} dz_k dw_k q(z_k)q(w_k)\right) \delta\left(s - \sum_{k=1}^{S-2} z_k w_k\right) \delta(Z\cos(\eta) + dt - s)\ ,
\end{aligned} \tag{99}$$

where $q(z)$ is the marginal distribution of $q(x,y)$:

$$q(z) = \int \mathrm{d}x\ q(x,z) = \int \mathrm{d}x\ q(z,x). \tag{100}$$

We can introduce the distribution of the sum:

$$q_s(t) = \int \mathrm{d}x\mathrm{d}\ yq(x,y)\delta(t-(x+y)). \tag{101}$$

The term

$$\int \left(\prod_{k=1}^{S-2} \mathrm{d}z_k \mathrm{d}w_k \; q(z_k) q(w_k) \right) \; \delta \left(s - \sum_{k=1}^{S-2} z_k w_k \right) \tag{102}$$

is the distribution of a sum of $S-2$ uncorrelated random variables. These random variables are the product zw of two random variables whose distribution is q. Since the second moment of $q(x)$ is finite, the central limit theorem holds and this distribution converges, in the large S limit, to a Gaussian distribution with mean

$$S \int \mathrm{d}x \mathrm{d}y \; q(y) q(x) \; xy = S E_1^2 \tag{103}$$

and variance

$$S \left(\int \mathrm{d}x \mathrm{d}y \; q(y) q(x) \; (xy)^2 - E_1^2 \right) = S E_2^4 \, . \tag{104}$$

We have therefore

$$P(\eta) = Z |\sin(\eta)| \int \mathrm{d}t \mathrm{d}s \; q_s(t) \frac{\exp\left(\frac{-(s - S E_1^2)^2}{2 S E_2^4} \right)}{\sqrt{2 S \pi} E_2^2} \delta(Z \cos(\eta) + dt - s) = (S(E_1^2 + E_2^2) - d) \\ \times \frac{|\sin(\eta)|}{\sqrt{2 S \pi} E_2^2} \int \mathrm{d}t \; q_s(t) \exp\left(- \frac{\left(S(E_1^2 + E_2^2)\cos(\eta) - d\cos(\eta) - S E_1^2 + dt \right)^2}{2 S E_2^4} \right) \, . \tag{105}$$

The distribution of η is not universal as it depends on $q_s(t)$, which depends on the distribution of the coefficients. On the other hand, the dependence is explicit, and it is possible to calculate $P(\eta)$ for any distribution $q(x, y)$.

We show explicitly the case of $q(x, y)$ being a bivariate normal distribution, i.e.,

$$q(x, y) = \frac{1}{2\pi E_2^2 \sqrt{1 - E_c^2}} \exp\left(- \frac{(x - E_1)^2 + (y - E_1)^2 - 2 E_c (x - E_1)(y - E_1)}{2 E_2^2} \right) \, . \tag{106}$$

In this case $q_s(t)$ is a normal distribution, and can be obtained from eq 101

$$\begin{aligned} q_s(t) &= \frac{1}{2\pi E_2^2\sqrt{1-E_c^2}} \int \mathrm{d}y \exp\left(-\frac{(t-y-E_1)^2+(y-E_1)^2-2E_c(t-y-E_1)(y-E_1)}{2E_2^2}\right) \\ &= \exp\left(-\frac{(1-E_c)(t-2E_1)^2}{4E_2^2}\right)\frac{1}{2\sqrt{\pi}E_2(1+E_c)\sqrt{1-E_c}} \,. \end{aligned} \tag{107}$$

Substituting into equation 105, we see that $P(\eta)$ has the form of a convolution of two Gaussians, and turns out to be equal to

$$P(\eta) = \frac{|\sin(\eta)|}{\sqrt{2\pi}\,\mathrm{var}(\cos(\eta))} \exp\left(-\frac{\big(\cos(\eta)-\langle\cos(\eta)\rangle\big)^2}{2\,\mathrm{var}(\cos(\eta))}\right) \,. \tag{108}$$

The mean $\langle\cos(\eta)\rangle$ and variance $\mathrm{var}(\cos(\eta))$ will be computed in the next section in the most general case of an arbitrary interaction distribution.

Supplementary Note 10.2 Moments for random matrices As explained in the previous section, the distribution of the side lengths is not a universal quantity, as it depends on the distribution of interaction strengths. In this section we compute the mean and the variance in the general case, showing that they depends only on E_1, E_2 and E_c.

Here and in the main text we do not report the moments of the side length η, but the moments of its cosine. The cosine of the side length measures the overlap between two rows of the interaction matrix (or the scalar product of two generators of the convex polytope). As its value gets close to one, the side length approaches zero.

Starting from equation 95, we have that

$$\big\langle \cos(\eta)\big\rangle = \frac{1}{S(S-1)}\sum_{i\neq j}\cos(\eta_{ij}) = \frac{1}{S(S-1)}\sum_{i\neq j}\left(\frac{\sum_k A_{ik}A_{jk}}{\sqrt{\sum_k A_{ik}A_{ik}\sum_l A_{jl}A_{jl}}}\right) \,, \tag{109}$$

Since we are interested in the large S limit, we can write the denominator as in equation 98 and

obtain

$$\left\langle \cos(\eta) \right\rangle = \frac{1}{S(S-1)} \sum_{i \neq j} \left(\frac{\sum_k A_{ik} A_{jk}}{-d + S(E_1^2 + E_2^2)} \right) \,, \tag{110}$$

and then

$$\left\langle \cos(\eta) \right\rangle = \frac{1}{S(S-1)} \sum_{i \neq j} \left(\frac{A_{ii} A_{ji} + A_{ij} A_{jj} + \sum_{k \neq i,j} A_{ik} A_{jk}}{-d + S(E_1^2 + E_2^2)} \right) \,. \tag{111}$$

In the large S limit, this becomes

$$\left\langle \cos(\eta) \right\rangle = \frac{-2dE_1 + SE_1^2}{-d + (S-2)(E_1^2 + E_2^2)} \tag{112}$$

to leading order in S.

In a similar way, we can write the second moment as

$$\left\langle \cos(\eta)^2 \right\rangle = \frac{1}{S(S-1)} \sum_{i \neq j} \cos(\eta_{ij})^2 = \frac{1}{S(S-1)} \sum_{i \neq j} \left(\frac{\sum_k A_{ik} A_{jk}}{\sqrt{\sum_k A_{ik} A_{ik} \sum_l A_{jl} A_{jl}}} \right)^2 \,. \tag{113}$$

In the large S limit we obtain

$$\begin{aligned} \left\langle \cos(\eta)^2 \right\rangle &= \frac{1}{S(S-1)} \sum_{i \neq j} \frac{\left(\sum_k A_{ik} A_{jk}\right)^2}{\left(-d + S(E_1^2 + E_2^2)\right)^2} = \frac{1}{S(S-1)} \sum_{i \neq j} \frac{\sum_k \sum_l A_{ik} A_{jk} A_{il} A_{jl}}{\left(-d + S(E_1^2 + E_2^2)\right)^2} \\ &= \frac{1}{S(S-1)} \sum_{i \neq j} \frac{\left(A_{ii} A_{ji} + A_{ij} A_{jj} + \sum_{k \neq i,j} A_{ik} A_{jk}\right)\left(A_{ii} A_{ji} + A_{ij} A_{jj} + \sum_{l \neq i,j} A_{il} A_{jl}\right)}{\left(-d + S(E_1^2 + E_2^2)\right)^2} \\ &= \frac{1}{S(S-1)} \sum_{i \neq j} \frac{d^2 (A_{ij} + A_{ji})^2 - 2d(A_{ij} + A_{ji}) \sum_{k \neq i,j} A_{ik} A_{jk} + (\sum_{k \neq i,j} A_{ik} A_{jk})^2}{\left(-d + S(E_1^2 + E_2^2)\right)^2} \,. \end{aligned} \tag{114}$$

We can compute the averages of the different terms, obtaining

$$\begin{aligned} \frac{1}{S(S-1)} \sum_{i \neq j} (A_{ij} + A_{ji})^2 &= \frac{1}{S(S-1)} \sum_{i \neq j} (A_{ij}^2 + A_{ji}^2 + 2A_{ji} A_{ji}) \\ &= 2(E_1^2 + E_2^2) + 2(E_c E_2^2 + E_1^2) = 4E_1^2 + 2(1 + E_c) E_2^2 \,, \end{aligned} \tag{115}$$

$$\frac{1}{S(S-1)}\sum_{i\neq j}(A_{ij}+A_{ji})\sum_{k\neq i\neq j}A_{ik}A_{jk} = \frac{1}{S(S-1)}\sum_{i\neq j}(A_{ij}+A_{ji})(S-2)E_1^2 \\ = 2(S-2)E_1^3\,, \tag{116}$$

and

$$\frac{1}{S(S-1)}\sum_{i\neq j}\left(\sum_{k\neq i\neq j}A_{ik}A_{jk}\right)^2 = \frac{1}{S(S-1)}\sum_{i\neq j}\sum_{k\neq i,j}\sum_{l\neq i,j}A_{ik}A_{il}A_{jk}A_{jl} \\ = \frac{1}{S(S-1)}\sum_{i\neq j}\sum_{k\neq i,j}\left(\sum_{l\neq i,j,k}(A_{ik}A_{il}A_{jk}A_{jl}) + A_{ik}^2A_{jk}^2\right) \\ = (S-2)(S-3)E_1^4 + (S-2)(E_1^2+E_2^2)^2\,. \tag{117}$$

We finally get that, in the large S limit,

$$\mathrm{var}(\cos(\eta)) = \left\langle \cos(\eta)^2\right\rangle - \left\langle \cos(\eta)\right\rangle^2 = \frac{2d^2(1+E_c)E_2^2 + S(E_2^2+E_1^2)^2 - SE_1^4}{\left(-d+S(E_1^2+E_2^2)\right)^2}\,. \tag{118}$$

Supplementary Note 11 Side heterogeneity for different structures and empirical networks

In figure 13 we considered the effect of four nonrandom structures on the mean and variance of the side lengths. The interaction strengths were drawn from a normal distribution with given mean, variance, and correlation. For some structures we considered multiple interaction types and therefore multiple means (one positive and one negative), in which case the coefficient of variation of the interactions and the correlation was constant and independent of the mean. Networks were parametrized as explained in section Supplementary Note 8.

- **Modular**. In this case we considered interaction matrices with a perfect block structure (to generate figure 3 we considered four blocks of equal size).
- **Bipartite**. In this case we considered an interaction matrix with two bipartite blocks of equal size. The mean interaction of the offdiagonal blocks was set to be negative, while the one of the in-diagonal blocks was positive.

- **Nested**. The interaction matrix had a bipartite structure. The diagonal blocks had a random structure with negative mean interaction strength. In the offdiagonal blocks, we consider a connectance equal to one half and we built a perfectly nested matrix. The mean interaction strength was positive in the offdiagonal blocks.

- **Cascade**. We build a matrix using the cascade model, and parameterize it with a positive and a negative mean depending on the role of the species in the interaction.

In the case of empirical structures, figure 3 of the main text, was obtained considering the same networks and the same parameterizations considered in section Supplementary Note 8. We compared $\text{var}(\cos(\eta))$ with the values expected in the random case. Figure 14 shows the comparison between $\left\langle \cos(\eta) \right\rangle$ obtained for empirical networks with the null prediction. Its value is well predicted by the null expectation for mutualistic networks, while the null expectations underestimates this value for food webs. This is consistent with the fact that the size of feasibility domain of random networks is larger that the one of empirical networks.

Supplementary Note 12 Feasibility domain for $S = 3$

When $S = 3$, it is possible to visualize in three dimensions a convex polyhedral cone and the feasibility domain [1]. In figure 15 we show a convex polyhedral cone in three dimensions and its generators.

An important feature of convex polyhedral cones is that if $\boldsymbol{r}$ belongs to the cone, then so does $c\boldsymbol{r}$ for any positive constant c. As explained in section Supplementary Note 3, this is a consequence of the linearity of equation 1. It is relevant therefore to limit our analysis to the growth rate vectors

on the unit sphere, i.e., to vectors $\boldsymbol{r}$ such that

$$\|\boldsymbol{r}\| = \sqrt{r_1^2 + r_2^2 + r_3^2} = 1 \ . \tag{119}$$

When we consider the vector in the feasibility domain on the surface of a unit sphere we obtain the areas of figure 1 in the main text. In this case, the quantity Ξ is the area of the triangle, while the side lengths are the three sides of the triangle. Note that the polygon is not a triangle (as it lies on a sphere), but rather a spherical triangle. Its sides are arcs of a circumference, while its corners are identified by the three generators of the convex polyhedral cone.

In the $S = 3$ case it is possible to obtain a closed expression for the area Ξ [11]:

$$\Xi = \frac{8}{\pi} \arctan\Big(\frac{|\det(\boldsymbol{G})|}{1 + \boldsymbol{g}^1 \cdot \boldsymbol{g}^2 + \boldsymbol{g}^2 \cdot \boldsymbol{g}^3 + \boldsymbol{g}^1 \cdot \boldsymbol{g}^3} \Big) + \Theta\Big(-1 - \boldsymbol{g}^1 \cdot \boldsymbol{g}^2 - \boldsymbol{g}^2 \cdot \boldsymbol{g}^3 - \boldsymbol{g}^1 \cdot \boldsymbol{g}^3 \Big) \ , \tag{120}$$

where the second term adds one to the first term when the argument of the arctangent is negative, while the matrix $\boldsymbol{G}$ is defined as

$$G_{ij} = \boldsymbol{g}^i \cdot \boldsymbol{g}^j \ . \tag{121}$$

Equation 120 can be expressed directly in terms of the matrix $\boldsymbol{A}$ using equation 12.

Supplementary Note 13 Nonlinear per capita growth rates

In general, the effect of a species on the per capita growth rate of other species is not linear. Equation 1 assumes this to be linear and the results presented in this paper were obtained under this assumption. Nonlinearity of the per capita growth rates can be thought of as a dependence of the interaction matrix $\boldsymbol{A}$ on $\boldsymbol{n}$:

$$\frac{\mathrm{d}n_i}{\mathrm{d}t} = n_i \left(r_i + \sum_{j=1}^{S} A_{ij}(\boldsymbol{n}) n_j \right) \ . \tag{122}$$

For instance, in the case of predator-prey interactions with a Holling type II functional response, it would have the form

$$A_{ij}(\boldsymbol{n}) = \frac{A^0_{ij}}{1 + \sum_j h_{ij} A^0_{ij} n_j} , \tag{123}$$

where the h_{ij} are the handling times.

The presence of nonlinearity has strong consequences for both feasibility and stability. It is no longer possible to disentangle feasibility and stability with a simple condition on A^0_{ij}. This means that feasibility will depend not only on the direction of $\boldsymbol{r}$, but also on its length.

The results presented here are a necessary stepping stone for assessing the feasibility of nonlinear systems. When the degree of nonlinearity is small (e.g., $h_{ij} \approx 0$), one can use our results, valid for the case $h_{ij} = 0$, to find the center of the feasibility domain and the generators. One can then treat the departure from $h_{ij} = 0$ as a small perturbation, and therefore, instead of having to explore the full vast parameter space, use the solution of the linear case as a starting point for numerical calculations to converge on the actual, nonlinear feasibility domain. On the other hand, in the limit of very large h_{ij} values, It is possible to show that the nonlinear form in equation 123 is approximately linear, and so again it is possible to use our method. The effect of intermediate values of h_{ij} on the feasibility domain is, however, still an open question.

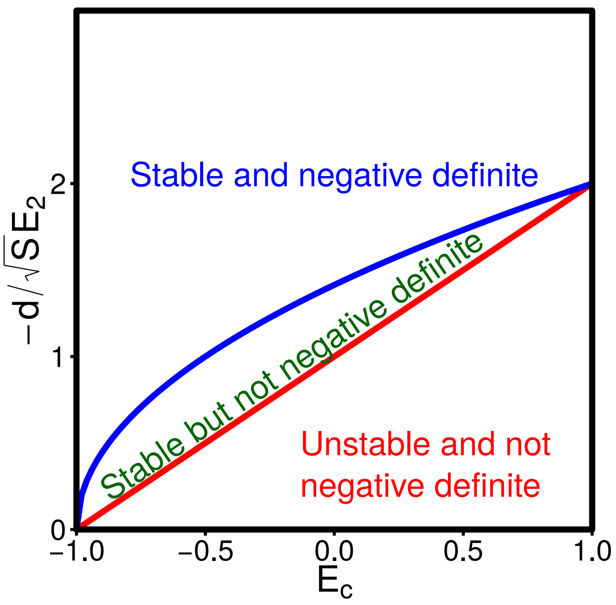

Supplementary Figure 1: Negative definiteness and stability for random matrices in the case $E_1 = 0$. The red curve describes the condition for stability (equation 45), while the blue curve corresponds to the negative definiteness condition (equation 46). The region above the blue curve corresponds to matrices that are both stable and negative definite, while the region below the red curve corresponds to unstable and non-negative definite matrices. The parameterizations that may still lead to stable and feasible points but we are not considering are in the region between the two curves. The shape of this region does not change substantially if S and E_2 are changed or if $E_1 < 0$. For $E_1 > 0$ the not negative definite but stable region is always smaller and eventually disappears (i.e., the blue and the red curve become the same) when S is large enough.

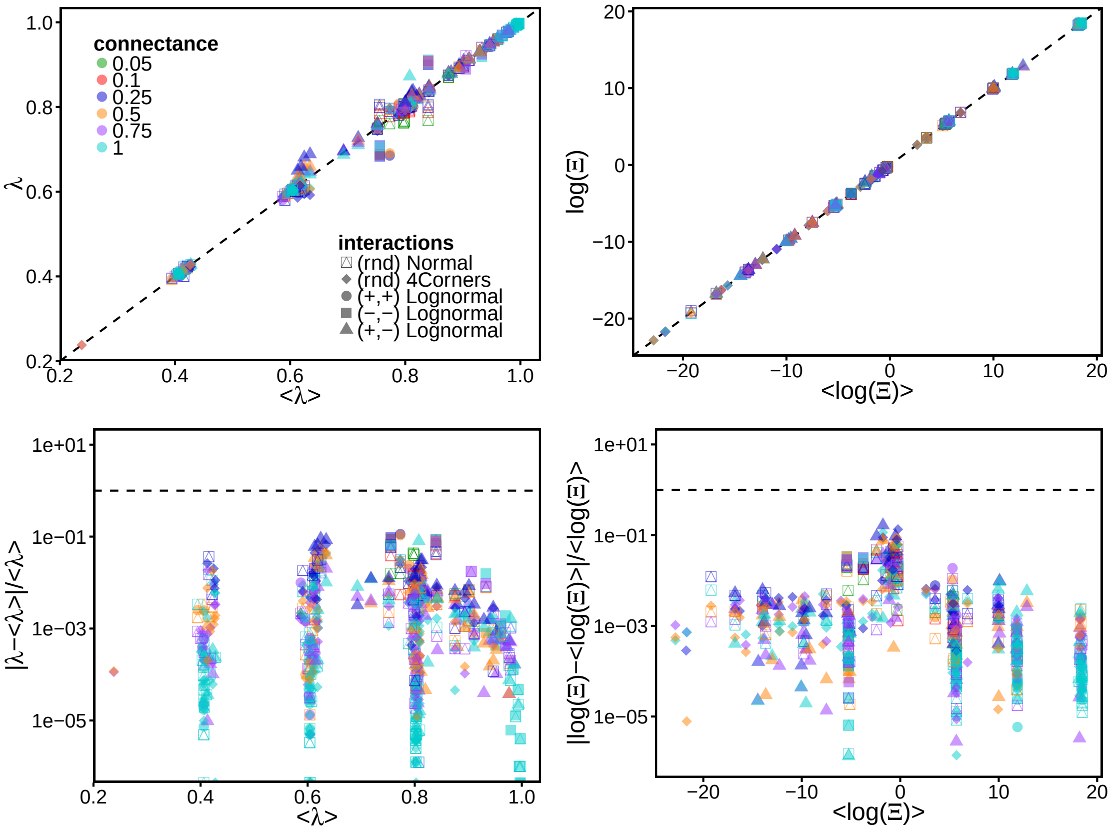

Supplementary Figure 2: Universality of λ and Ξ in random matrices. The two left panels refer to the eigenvalue with the largest real part λ of the interaction matrix $\boldsymbol{A}$, while the right ones to the size Ξ of the feasibility domain. We consider different values of the connectance (colors) and different distributions (shape), such that there were multiple combination of connectances and distributions having the same values of E_1, E_2, and E_c. We computed the averages $\langle \lambda \rangle$ and $\langle \log(\Xi) \rangle$ over all realizations of the matrices having the same values of E_1, E_2, and E_c. If the value of λ and Ξ are universal, then they depend only on E_1, E_2, and E_c, and therefore their values are equal to the mean: universality holds if $\lambda = \langle \lambda \rangle$ and $\log(\Xi) = \langle \log(\Xi) \rangle$. The top panels show that these two quantities are equal and the bottom panels quantify their deviations. We know that λ is universal, and since Ξ has a similar behavior, we conclude that Ξ is also universal.

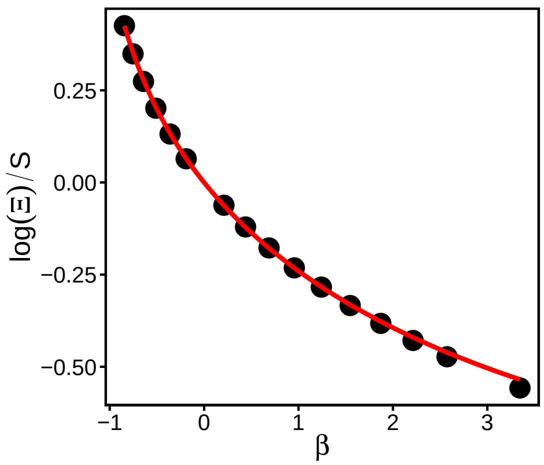

Supplementary Figure 3: Approximation of Ξ using mean field theory. The black dots are numerical simulations obtained by integrating Ξ numerically (see section Supplementary Note 4) for a constant interaction matrix. The red curve is the analytical approximation obtained using the mean-field approximation (see equation 81). β is a function of E_1 and S, and is defined in equation 64. The range of β considered here is the same of the one appearing in figure 1 of the main text.

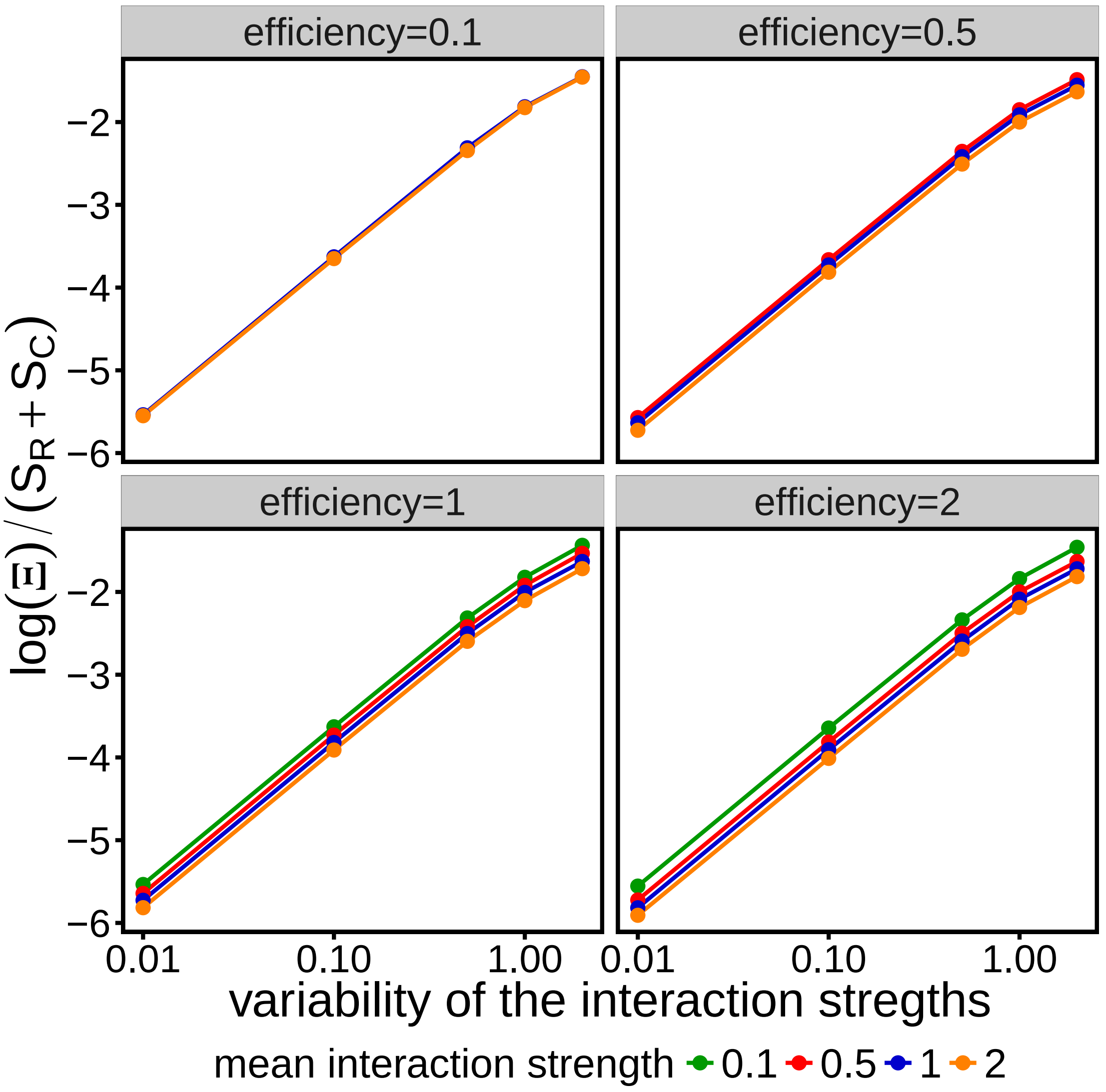

Supplementary Figure 4: Feasibility domain of consumer-resource community. We considered an interaction matrix of the form of equation 84, with $S_R = 40$ and $S_C = 30$, $C_{ij} = 1$ if $i = j$ and zero otherwise, $Z_i W_j = \eta > 0$ for any i and j, and $\boldsymbol{B}$ with entries independently drawn from a Lognormal distribution with mean μ and variance $c_v^2 \mu^2$. For each parameterization we computed the feasibility domain Ξ using the method explained in section Supplementary Note 4. The value of Ξ is mostly determined by the coefficient of variation of the interaction, and it depends only weakly on the mean interaction strength μ and the efficiency η.

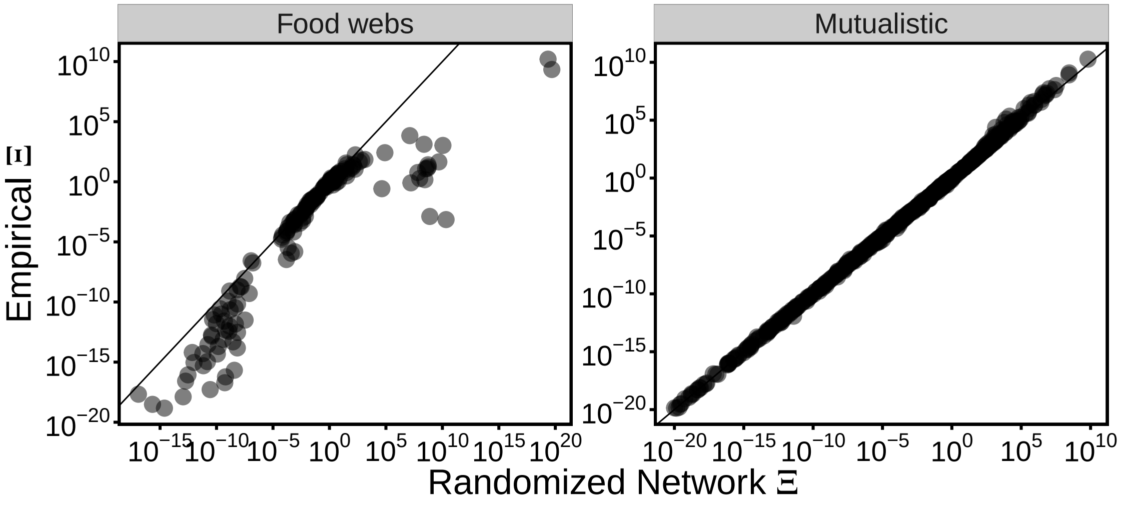

Supplementary Figure 5: Size of feasibility domain Ξ in empirical networks and randomizations. Empirical networks and their randomizations were parametrized as explained in section Supplementary Note 8. Each empirical network was parametrized 100 times and the average Ξ was compared with the one obtained by averaging 100 randomizations. Each point in this plot correspond therefore to a value of Ξ of an empirical network and its randomizations averaged over the extraction of the interaction strenghts for a given combination of the parameters as explained in section Supplementary Note 8.

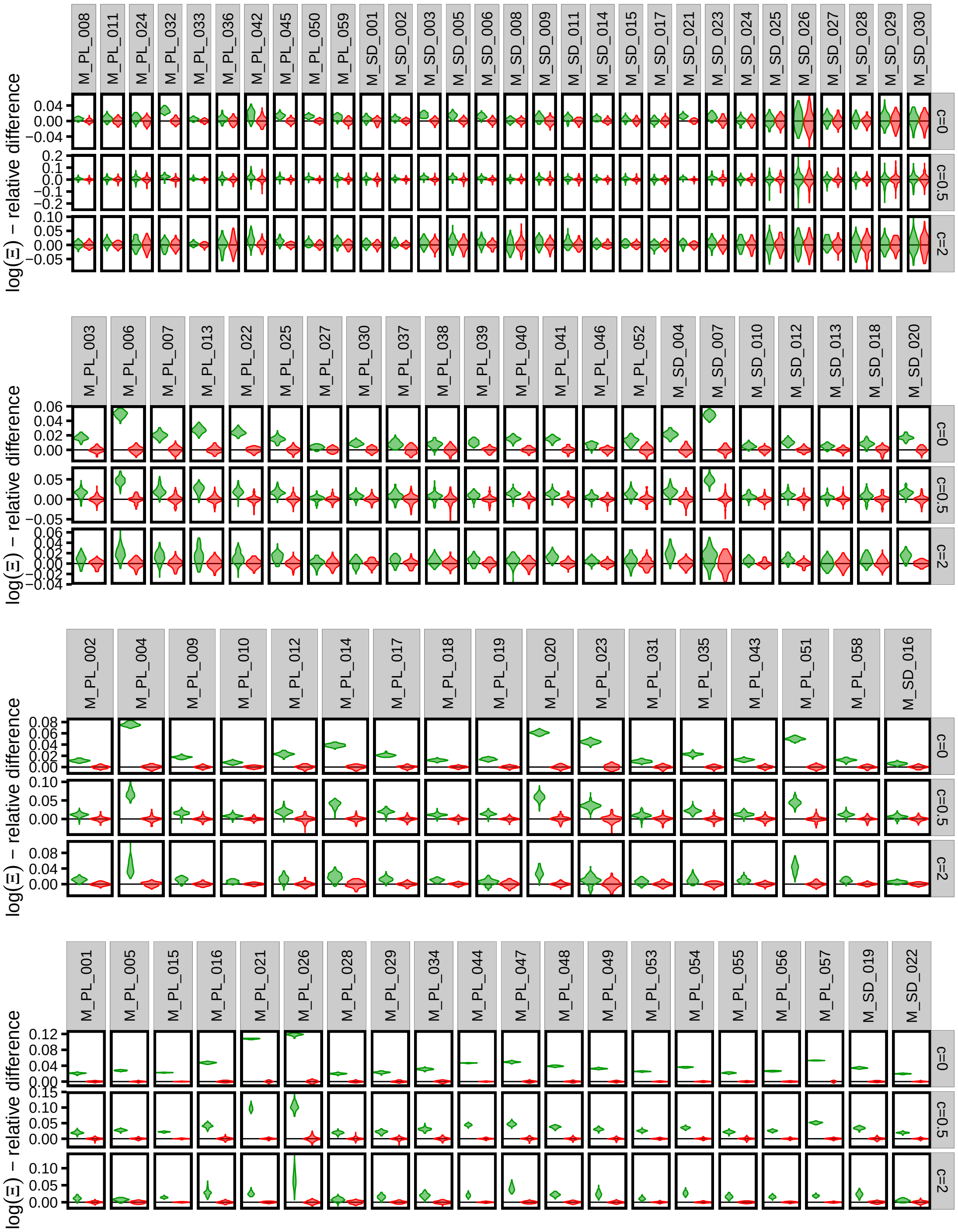

Supplementary Figure 6: We measure the effect of mutualistic network structure on the size of the feasibility domain as described in section Supplementary Note 9.1. Red violin plots are randomizations, green ones are empirical networks. The empirical networks are grouped in four rows based on the number of species ($S < 50$, $50 \leq S < 80$, $80 \leq S < 150$ and $S \geq 150$, respectively). This figure was obtained with $\mu_+ = 0.25\mu_{max}$, $\mu_- = 0$ and for three different values of c.

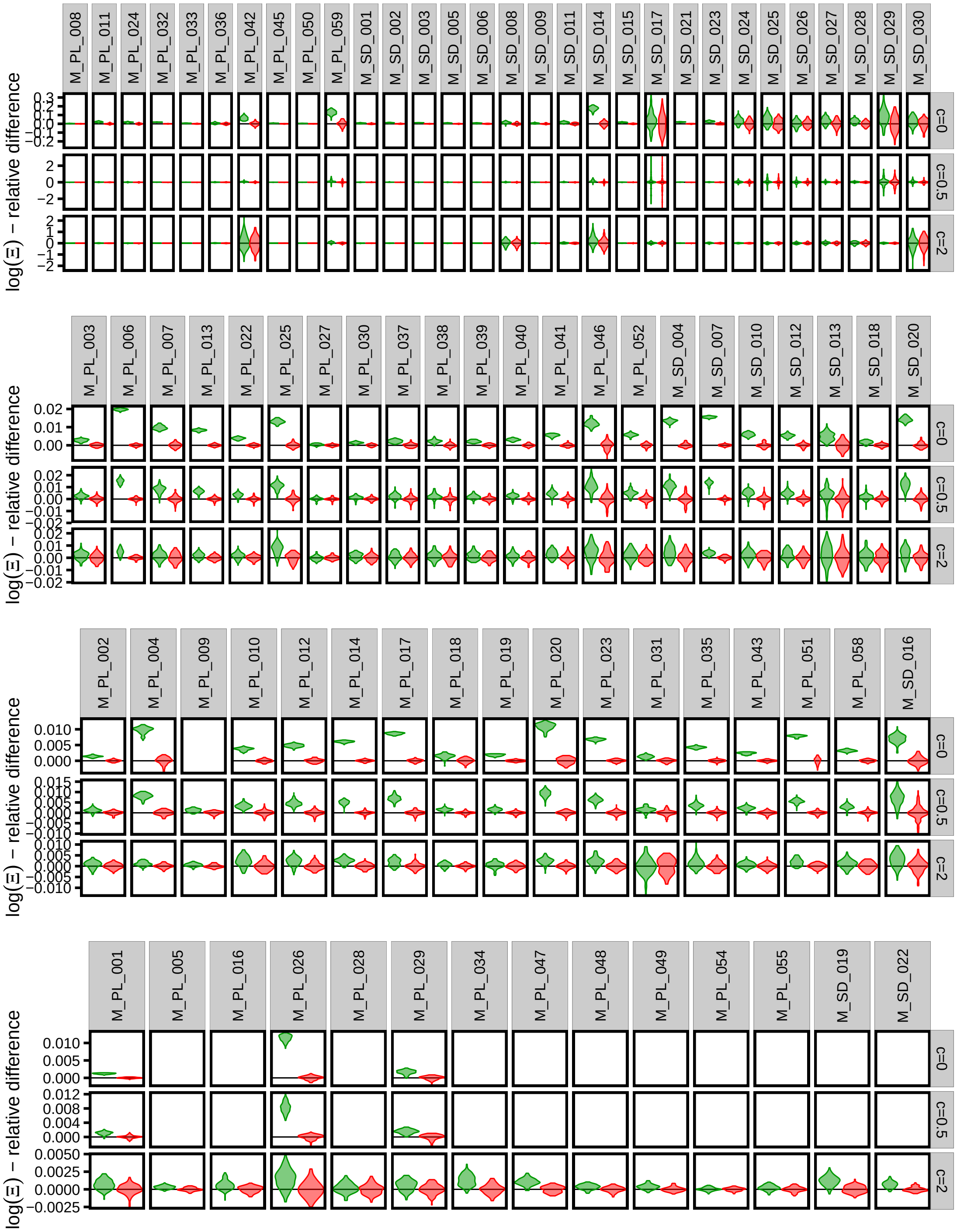

Supplementary Figure 7: Same as figure 6 but with $\mu_+ = 0.25\mu_{max}$ and $\mu_- = 0.5\mu_+$

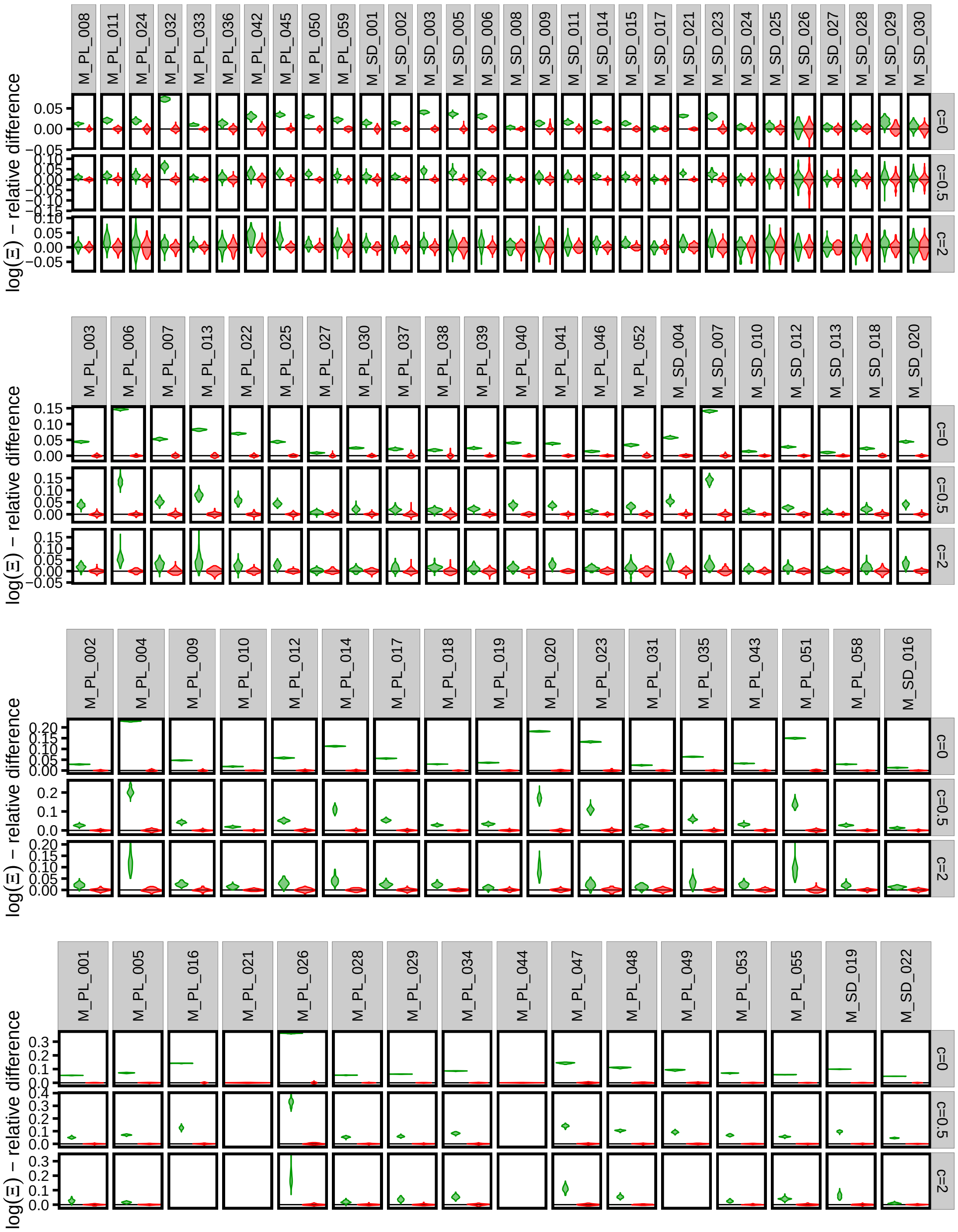

Supplementary Figure 8: Same as figure 6 but with $\mu_+ = 0.5\mu_{max}$ and $\mu_- = 0$

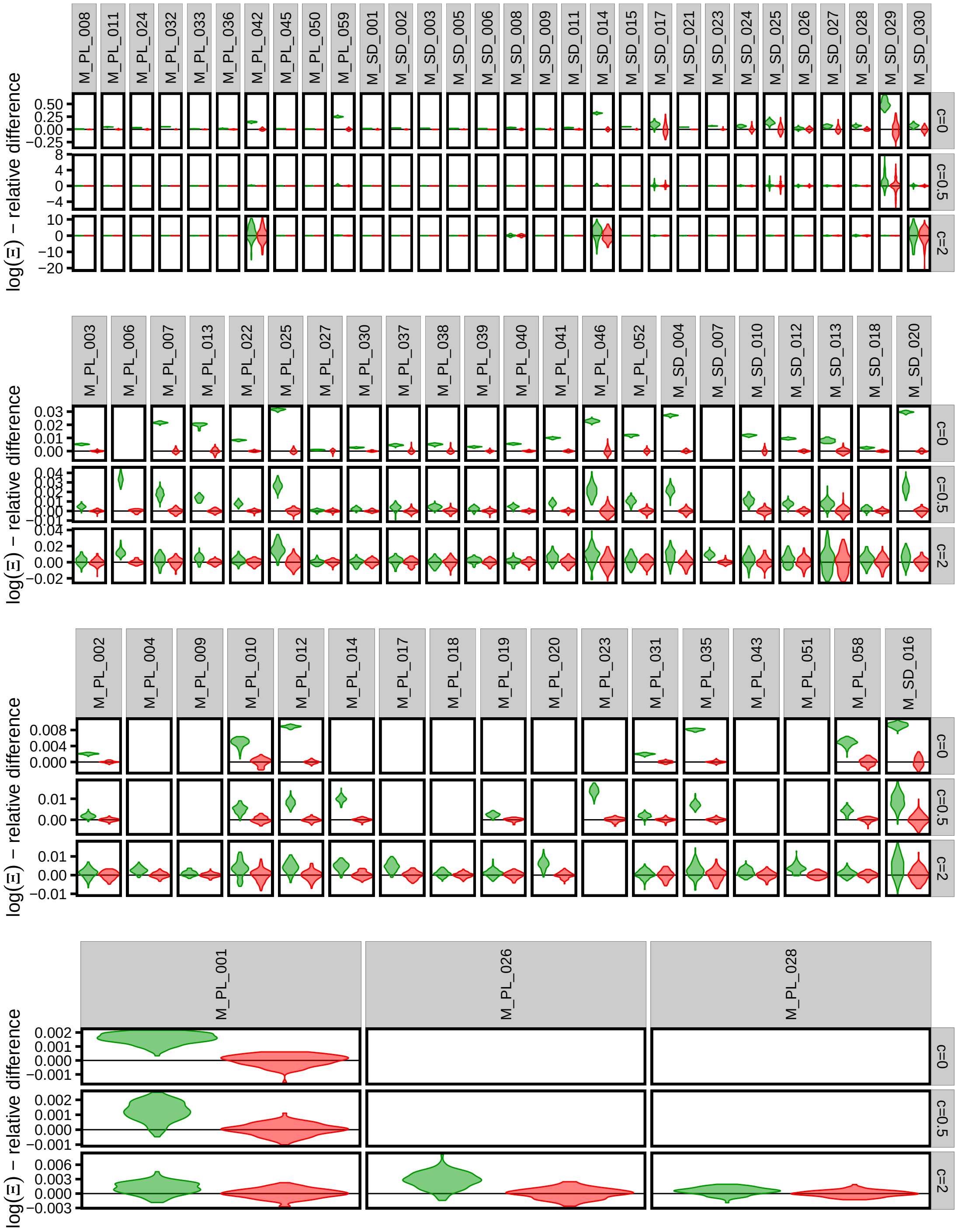

Supplementary Figure 9: Same as figure 6 but with $\mu_+ = 0.5\mu_{max}$ = $\mu_- = 0.5\mu_+$

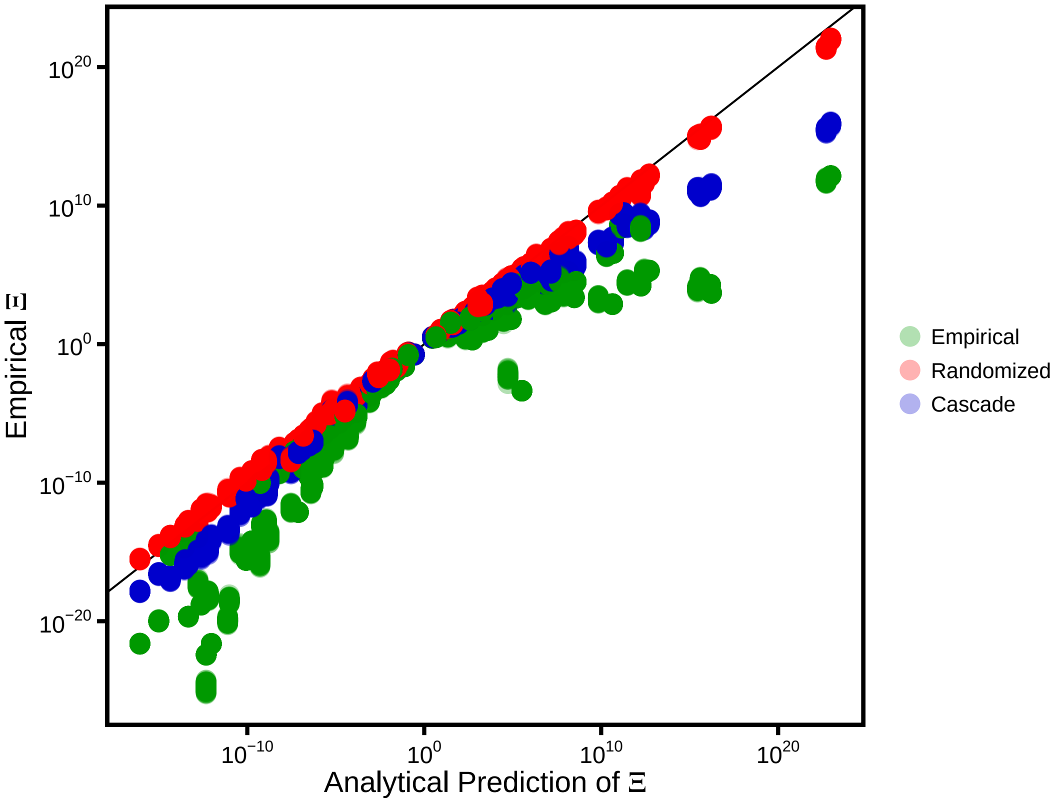

Supplementary Figure 10: In this figure we compared the analytical prediction of the feasibility domain obtained in section Supplementary Note 6 with the numerical calculated values for random networks, empirical networks and networks generated via the cascade models. The feasibility domain of random networks is well predicted by our analytical approximation, which fails to predict the empirical one and the one obtained using the cascade model.

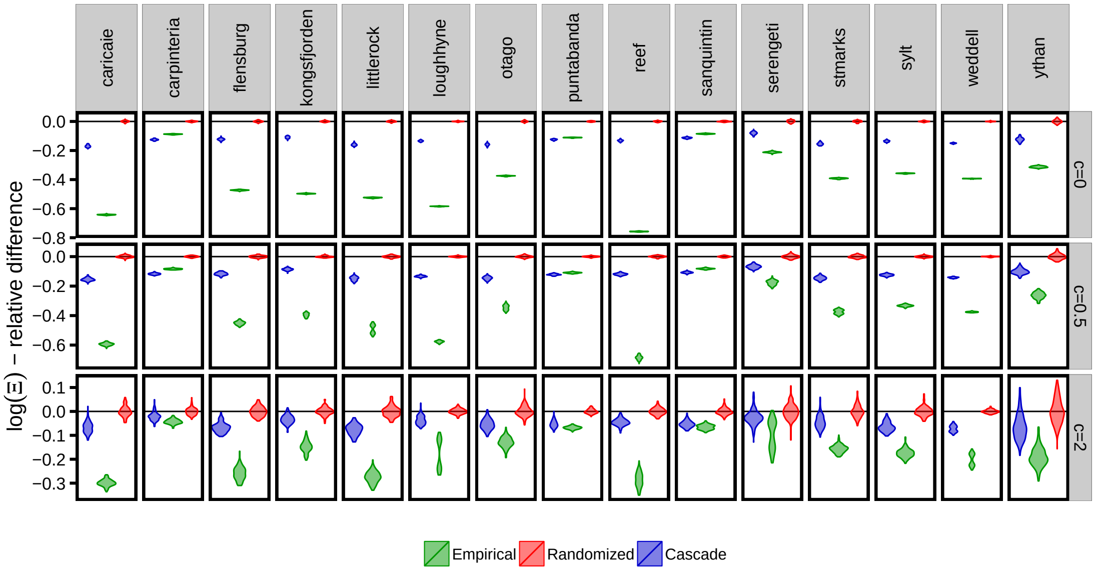

Supplementary Figure 11: We measure the effect of food web network structure on the size of the feasibility domain as described in section Supplementary Note 9.2. Red violin plots are randomizations, green ones are empirical networks, while blue ones correspond to the cascade model. This figure was obtained as explained in section Supplementary Note 9.2 with $\mu_- = 0.25\mu_{max}$, $\mu_+ = 0.5\mu_-$ and for three different values of c.

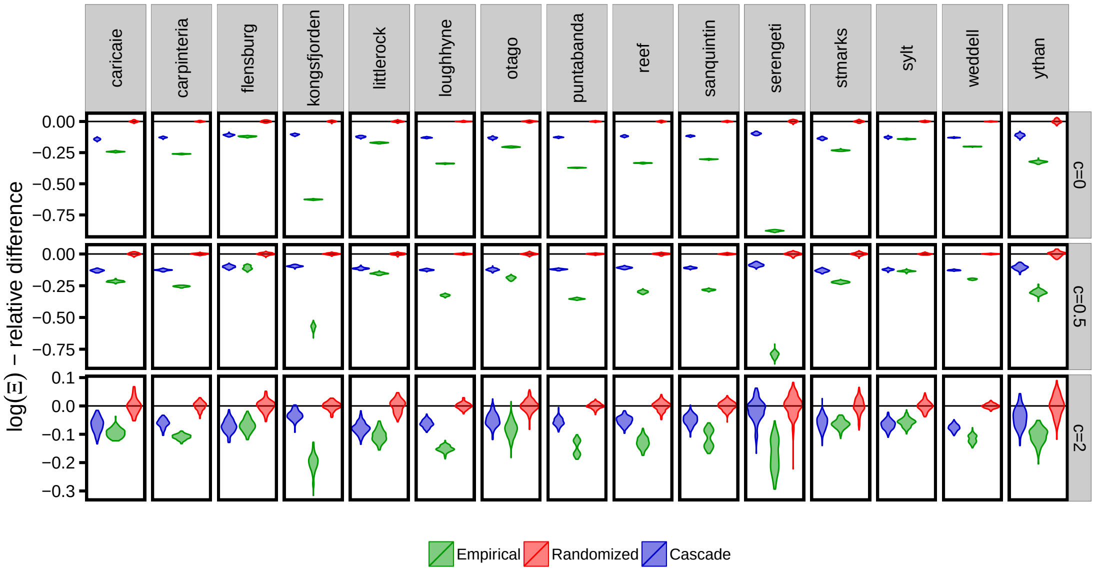

Supplementary Figure 12: Same as figure 12 but with $\mu_{-} = 0.25\mu_{max}$ and $\mu_{+} = 2\mu_{-}$

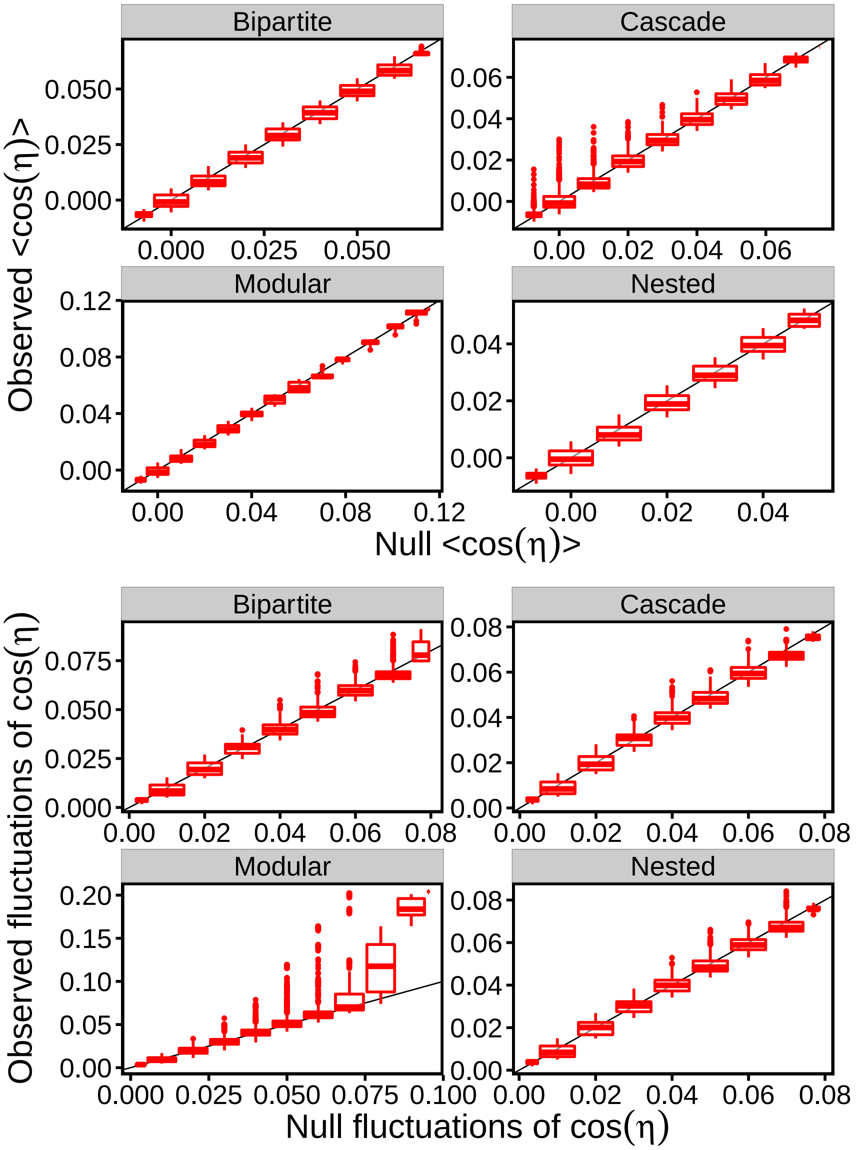

Supplementary Figure 13: We measure the effect of non random structures of mean and variance of side lengths. With the exception of the cascade model, all the structures considered do not have an important effect on $\langle\cos(\eta)\rangle$. On the other side, all the non-random structures considered have a positive effect on the variance of $\cos(\eta)$. All the networks considered had a connectance $C = 0.2$.

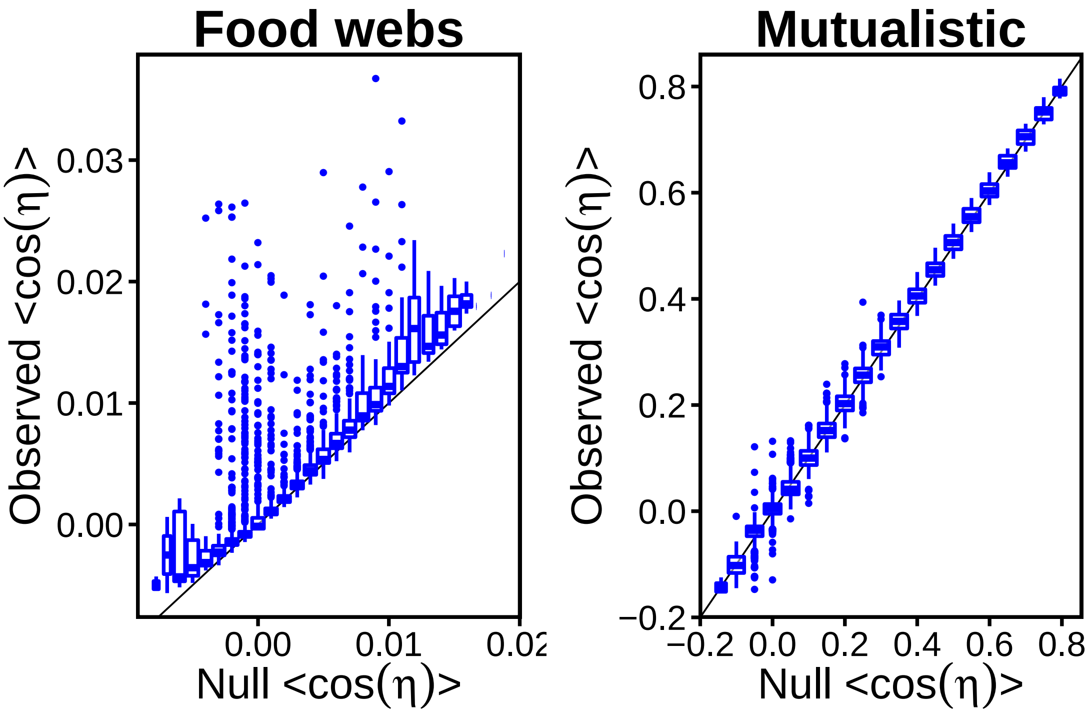

Supplementary Figure 14: Comparision between $\langle \cos(\eta) \rangle$ obtained for empirical networks and its null expectation for empirical food webs and mutualistic networks. This figure was realized with the same parametrization of figure 3 of the main text and as described in section Supplementary Note 8.

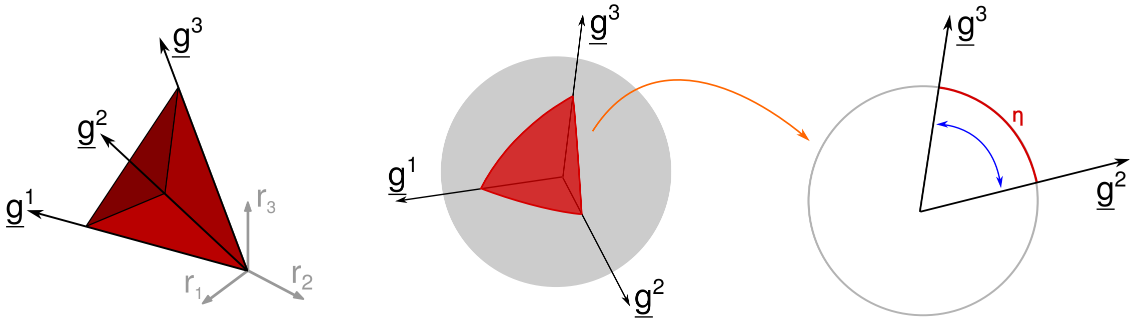

Supplementary Figure 15: Convex polyhedral cone and its section on a sphere. Left: the feasibility domain is a convex polyhedral cone, which is completely determined by its S generators (when $S = 3$ we have 3 generators $\boldsymbol{g}^1$, $\boldsymbol{g}^2$, and $\boldsymbol{g}^3$). Center: since we consider a linear equation we can focus the analysis only on the intersection between the convex polyhedral cone and the unit sphere's surface, which in three dimensions results in a spherical triangle. Right: each side of the convex polyhedral cone can be determined from a pair of generators as an arc η of the sphere's surface. Since we are considering the unit sphere, the arc length η is equal to the angle between the two generators.

Supplementary Table 1: References and properties of the 15 food webs analyzed in the work

Name	S	Number of links	Connectance
Ythan Estuary [25]	92	414	0.1
St. Marks [26]	143	1763	0.17
Grande Cariçaie [27]	163	2048	0.16
Serengeti [28]	170	585	0.04
Flensburg Fjord [29]	180	1567	0.1
Otago Harbour [30]	180	1856	0.12
Little Rock Lake [31]	181	2316	0.14
Sylt tidal basin [32]	230	3298	0.12
Caribbean Reef [33]	249	3293	0.11
Kongs Fjorden [34]	270	1632	0.04
Carpinteria Salt Marsh [35]	273	3878	0.1
San Quintin [35]	290	3934	0.09
Lough Hyne [36]	349	5088	0.08
Punta Banda [35]	356	5291	0.09
Weddell Sea [37]	488	15435	0.13

Feasibility and coexistence of large ecological networks: a geometrical approach
Supplementary References

1. Yu M. Svirezhev and Dmitrii Olegovich Logofet. *The stability of biological communities*. Nauka, Moscow, 1978.

2. Eugenius Kaszkurewicz and Amit Bhaya. *Matrix Diagonal Stability in Systems and Computation*. Birkhäuser Boston, Boston, MA, 2000.

3. C. R. Johnson. Positive Definite Matrices. *The American Mathematical Monthly*, 77(3):259, mar 1970.

4. Abraham Berman and Daniel Hershkowitz. Matrix diagonal stability and its implications. *SIAM Journal on Algebraic Discrete Methods*, 4(3):377–382, 1983.

5. B. S. Goh. Global Stability in Many-Species Systems, 1977.

6. R. P. Rohr, S. Saavedra, and Jordi Bascompte. On the structural stability of mutualistic systems. *Science*, 345(6195):1253497–1253497, July 2014.

7. Vito Volterra. *Leçons sur la théorie mathématique de la lutte pour la vie*. J. Gabay, 1931.

8. Si Tang and Stefano Allesina. Reactivity and stability of large ecosystems. *Frontiers in Ecology and Evolution*, 2:21, jun 2014.

9. R. Tyrrell Rockafellar. *Convex Analysis*. Princeton University Press, 1997.

10. Jason M. Ribando. Measuring Solid Angles Beyond Dimension Three. *Discrete & Computational Geometry*, 36(3):479–487, oct 2006.

11. Daniel Gourion and Alberto Seeger. Deterministic and stochastic methods for computing volumetric moduli of convex cones. *Computational & Applied Mathematics*, 29(2):215–246, 2010.

12. Robert M. May. Will a Large Complex System be Stable? *Nature*, 238(5364):413–414, August 1972.

13. Stefano Allesina and Si Tang. Stability criteria for complex ecosystems. *Nature*, February 2012.

14. Terence Tao, Van Vu, and Manjunath et al. Krishnapur. Random matrices: Universality of ESDs and the circular law. *The Annals of Probability*, 38(5):2023–2065, September 2010.

15. Terence Tao and Van Vu. Random Matrices: Universality of Local Eigenvalue Statistics up to the Edge. *Communications in Mathematical Physics*, 298(2):549–572, April 2010.

16. Terence Tao and Van Vu. Random matrices: Universality of local eigenvalue statistics. *Acta Mathematica*, 206(1):127–204, March 2011.

17. Stefano Allesina and Si Tang. The stabilitycomplexity relationship at age 40: a random matrix perspective. *Population Ecology*, 57(1):63–75, January 2015.

18. Stefano Allesina and Si Tang. The stabilitycomplexity relationship at age 40: a random matrix perspective. *Population Ecology*, 57(1):63–75, jan 2015.

19. Giorgio Parisi. *Statistical Field Theory*. Perseus Books, 1998.

20. T. H. Berlin and M. Kac. The Spherical Model of a Ferromagnet. *Physical Review*, 86(6):821–835, June 1952.

21. Ted J. Case and Richard G. RG Casten. Global stability and multiple domains of attraction in ecological systems. *The American Naturalist*, 113(5):705–714, may 1979.

22. Jan O Haerter, Namiko Mitarai, and Kim Sneppen. Food Web Assembly Rules for Generalized Lotka-Volterra Equations. *PLoS computational biology*, 12(2):e1004727, feb 2016.

23. J E Cohen, F Briand, and C M Newman. *Community food webs: Data and theory*. Springer-Verlag, Berlin, 1990.

24. Stefano Allesina, Jacopo Grilli, György Barabás, Si Tang, Johnatan Aljadeff, and Amos Maritan. Predicting the stability of large structured food webs. *Nature communications*, 6:7842, January 2015.

25. Joel E Cohen, Daniella N Schittler, David G Raffaelli, and Daniel C Reuman. Food webs are more than the sum of their tritrophic parts. *Proceedings of the National Academy of Sciences*, 106(52):22335–22340, 2009.

26. Robert R Christian and Joseph J Luczkovich. Organizing and understanding a winter's seagrass foodweb network through effective trophic levels. *Ecological Modelling*, 117(1):99–124, 1999.

27. Marie-France Cattin Blandenier. *Food web ecology: models and application to conservation*. PhD thesis, 2004.

28. Edward B Baskerville, Andy P Dobson, Trevor Bedford, Stefano Allesina, T Michael Anderson, and Mercedes Pascual. Spatial guilds in the Serengeti food web revealed by a Bayesian group model. *PLoS computational biology*, 7(12):e1002321, 2011.

29. C Dieter Zander, Neri Josten, Kim C Detloff, Robert Poulin, John P McLaughlin, and David W Thieltges. Food web including metazoan parasites for a brackish shallow water ecosystem in Germany and Denmark: Ecological Archives E092-174. *Ecology*, 92(10):2007, 2011.

30. Kim N Mouritsen, Robert Poulin, John P McLaughlin, and David W Thieltges. Food web including metazoan parasites for an intertidal ecosystem in New Zealand: Ecological Archives E092-173. *Ecology*, 92(10):2006, 2011.

31. Neo D Martinez. Artifacts or attributes? Effects of resolution on the Little Rock Lake food web. *Ecological Monographs*, 61:367–392, 1991.

32. David W Thieltges, Karsten Reise, Kim N Mouritsen, John P McLaughlin, and Robert Poulin. Food web including metazoan parasites for a tidal basin in Germany and Denmark: Ecological Archives E092-172. *Ecology*, 92(10):2005, 2011.

33. Silvia Opitz. *Trophic interactions in Caribbean coral reefs*. Number 1085. WorldFish, 1996.

34. Ute Jacob, Aaron Thierry, Ulrich Brose, Wolf E Arntz, Sofia Berg, Thomas Brey, Ingo Fetzer, Tomas Jonsson, Katja Mintenbeck, Christian Mollmann, and Others. The role of body size in complex food webs: A cold case. *Advances In Ecological Research*, 45:181–223, 2011.

35. Ryan F Hechinger, Kevin D Lafferty, John P McLaughlin, Brian L Fredensborg, Todd C Huspeni, Julio Lorda, Parwant K Sandhu, Jenny C Shaw, Mark E Torchin, Kathleen L Whitney, and Others. Food webs including parasites, biomass, body sizes, and life stages for three California/Baja California estuaries: Ecological Archives E092-066. *Ecology*, 92(3):791, 2011.

36. Jens O Riede, Ulrich Brose, Bo Ebenman, Ute Jacob, Ross Thompson, Colin R Townsend, and Tomas Jonsson. Stepping in {E}lton's footprints: a general scaling model for body masses and trophic levels across ecosystems. *Ecology Letters*, 14(2):169–178, 2011.

37. Ute Jacob. *Trophic dynamics of Antarctic shelf ecosystems: food webs and energy flow budgets*. PhD thesis, Bremen, Univ., Diss., 2005.
